# New insights into disordered proteins and regions according to the FOD-M model

**DOI:** 10.1371/journal.pone.0275300

**Published:** 2022-10-10

**Authors:** Irena Roterman, Katarzyna Stapor, Piotr Fabian, Leszek Konieczny

**Affiliations:** 1 Department of Bioinformatics and Telemedicine, Jagiellonian University, Medical College, Kraków, Poland; 2 Faculty of Automatic, Department of Applied Informatics, Electronics and Computer Science, Silesian University of Technology, Gliwice, Poland; 3 Faculty of Automatic, Electronics and Computer Science, Department of Algorithmics and Software, Silesian University of Technology, Gliwice, Poland; 4 Chair of Medical Biochemistry, Jagiellonian University, Medical College, Kraków, Poland; Weizmann Institute of Science, ISRAEL

## Abstract

A collection of *intrinsically disordered proteins* (IDPs) having regions with the status of *intrinsically disordered* (IDR) according to the Disprot database was analyzed from the point of view of the structure of hydrophobic core in the structural unit (chain / domain). The analysis includes all the *Homo Sapiens* as well as *Mus Musculus* proteins present in the DisProt database for which the structure is available. In the analysis, the fuzzy oil drop modified model (FOD-M) was used, taking into account the external force field, modified by the presence of other factors apart from polar water, influencing protein structuring. The paper presents an alternative to secondary-structure-based classification of *intrinsically disordered regions* (IDR). The basis of our classification is the ordering of hydrophobic core as calculated by the FOD-M model resulting in *FOD-ordered* or *FOD-unordered* IDRs.

## 1. Introduction

Many biologically active proteins fail to form unique three-dimensional structures under physiological conditions, either along their entire lengths or locally. These proteins are known as *intrinsically disordered proteins* (IDPs) or in the latter case—*intrinsically disordered regions* (IDRs) among several other names [1–4]. Determining the criteria for the classification of these proteins can be complex [5–7]. An important element in the classification of IDPs is the relationship between the presence of such fragments and the biological function performed by a given protein [7]. The presence of fragments or even entire proteins with IDP status is sometimes associated with the non-folding phenomenon [8,9].

IDPs are a challenge for methods predicting protein structure. This is the case when the available structure of the complex gives the form obtained by the protein when it interacts with another component of the complex. The form of the interface, which in the free protein takes the form of IDP, already represents a static form in the complex [10–12]. The participation of IDPs in the context of a specific biological process—often critical for functioning—is important [13,14]. The state of disorder is also associated with the molten globule state—a state obtained by partial unfolding or a state preceding the final collapse in the folding process [15]. The phenomenon of IDPs turns out to be of special importance in the context of the processes related to the amyloid transformation [16,17]. The availability of numerous databases is a facilitation for the analysis of IDPs: **FuzDB** [18,19], **Disprot** [20–24], **MOBIDB** [25,26], **IDEAL** [27–29].

The database used in our study, the Disprot, is a database collecting proteins with a recognized disordered element, along with a rich literature discussing the experimental basis for identification [20–24]. A valuable feature of this database is the extensive and accurate experimental documentation of the status of a given fragment of a chain, including the experimental technique identifying the status of the protein in question. So it is possible to verify every IDP and every IDR [20–24]. However, the topic of intrinsically disordered proteins seems to be recognized to the extent that allows the prediction of chain fragments meeting the classification criteria of this group of structures [30–32].

Available servers for the characterization of segments or whole proteins belonging to IDPs are:

**DISOselect–**comparing performance of 12 selected predictors [33–36]
**ANCHOR** 2 –predicting protein binding regions in disordered proteins [37,38]
**MoRFChibi**–oriented to the structural changes of the disorder-to-order transition occurring during processes related to biological function [39–41].

The present work characterizes IDPs and IDRs considering the environment treated as an external force field derived from polar water but also modified by the presence of other factors, in particular hydrophobic ones. For this purpose, a model called fuzzy oil drop (FOD) model and its modified version FOD-M was applied [42–44]. In these models, the degree of compliance of the hydrophobicity distribution in the protein with the idealized distribution–expressed with the use of a 3D Gauss function—is determined by the value of the parameter *RD*. On the other hand, the degree of participation of factors other than polar water is expressed by the value of the parameter *K*. These parameters are described in detail later in the Materials and Methods section.

Based on the mentioned two parameters from FOD-M model (*RD* and *K*), we propose the alternative to the secondary-structure-based classification of intrinsically disordered regions(as presented in Disprot database) that complements the existing one enabling a new, richer perspective paving the way for further research. *T*he basis of our classification is the ordering of hydrophobic core as calculated by FOD-M model resulting in *FOD-ordered* or *FOD-unordered* IDRs.

## 2. Results

Our method for determining the status of IDR with respect to its structural unit (chain or domain) of a given IDP relies on the values of parameters *RD* and *K* defined in the FOD-M model (described in Materials and Methods). This forms the basis of our assessment of the specificity of a given IDR as well as the proposed alternative classification of IDRs.

### 2.1. General characteristics of the analyzed IDPs based on the FOD-M model

The number of analyzed proteins is limited to those human and mouse proteins present in the DisProt database, the structure of which is available in the PDB database along with the solved structure of their IDRs [20–24]. Whole the analysis for mouse proteins is contained in the Supporting Information (S1 Appendix).

For a collection of 120 human proteins (see S1 Table in S1 Appendix) having the status IDP according to the Disprot database, the FOD-M model was constructed to enable the analysis of the structure of their hydrophobic cores. The calculated parameters of this model–*RD* and *K* for both the structural unit (chain / domain) as well as for its IDR were calculated according to the procedure described in Materials and Methods. The numerical results are presented in S2 Table in S1 Appendix.

On the basis of the obtained results (S2 Table in S1 Appendix), it is possible to define the characteristics of these proteins and their classification based on the FOD-M model.

**Fig 1:**
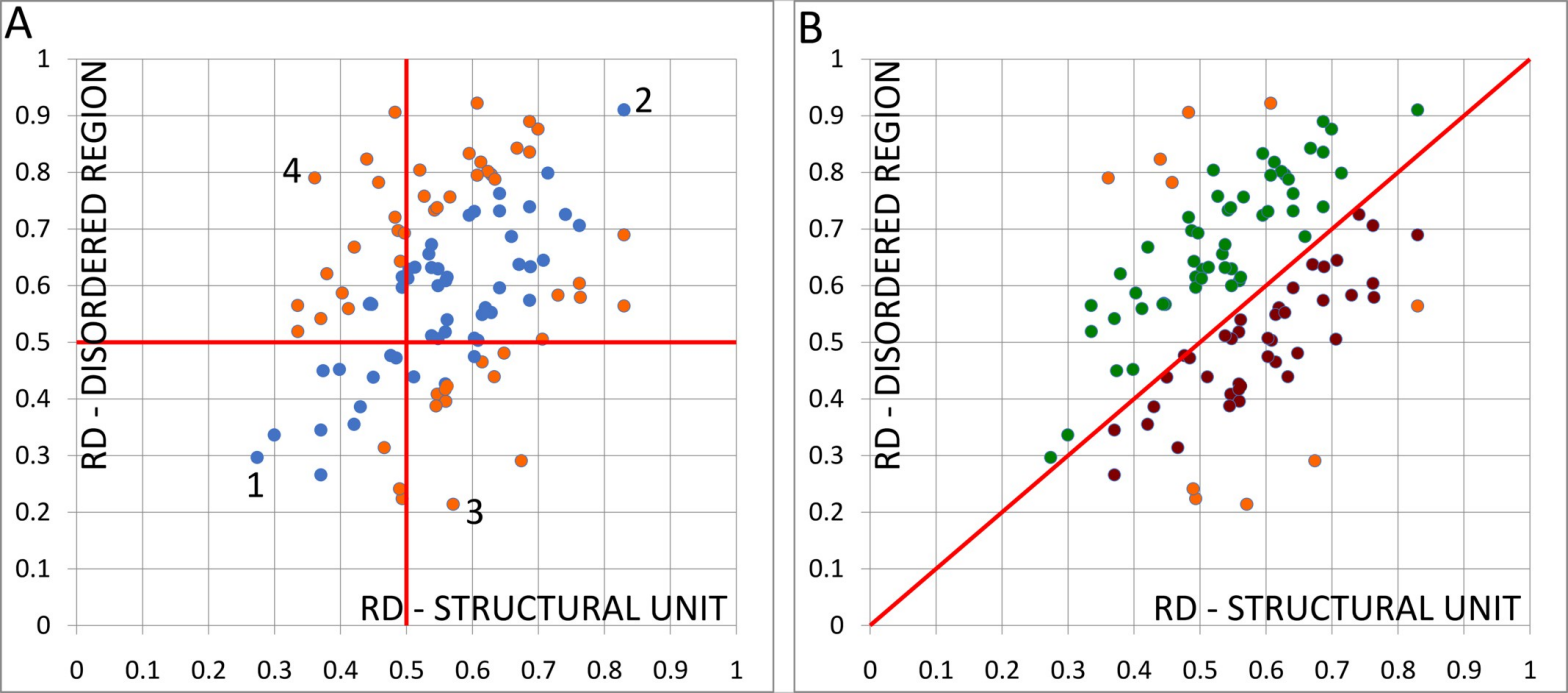
The scatterplots of the values of parameter *RD* for IDR and its structural unit (chain/domain) for the whole set of IDPs from S1 Table in S1 Appendix (detailed description–see text). A—(blue points “represent” the correlation coefficient 0.8). B–two subsets: The upper (green points) and the lower (red points) obtained by considering the location of points with respect to line *RD*_*IDR*_ = *RD*_*SU*_. The orange points in A and B correspond to the outstanding points.

The **s**catterplots of the values of the calculated parameter *RD* for the IDR and its structural unit (chain/domain) for the whole set of IDPs from S1 Table in S1 Appendix is presented in Fig 1A (blue and orange points). Looking at the scatter plot in Fig 1A, an approximately linear relationship between the parameters *RD* for the *structural unit* (SU) and its IDR can be observed.

The observed linear relationship was further investigated. The calculated Pearson correlation coefficient for the whole set of IDPs from the S1 Table in S1 Appendix is equal to 0.506. In order to investigate the observed regularity—a linear relationship between the RD parameters–the proteins with a significantly different status (measured by the relation of RD parameters) have been removed from the further analysis. However, a detailed analysis of the (probable) reasons for their discrepancy has been made below on the basis of the representatives from each quadrant of the coordinate system. Thus, the further selection has been performed as a stepwise elimination of the extreme points based on the visual inspection until the correlation coefficient reaches the value 0.8. Such obtained set is presented in Fig 1A by the blue points (the correlation coefficient for this group is equal to 0.799) while the remaining elements are marked as orange ones. Such a high value of a correlation means that the status of IDR is compatible with the status of the structural unit in which it occurs.

Using the similar method of stepwise elimination based on the visual inspection, the distribution of all points corresponding to the values of *RD* parameter for IDR and structural unit reveals the natural segmentation into the two groups–above and under–the line *RD*_*IDR*_ = *RD*_*SU*_ (see Fig 1B).

1. **Upper subset**: IDPs with a higher status for IDR versus the status of a structural unit with correlation coefficient = 0.865 (Fig 1B–marked as green points)
2. **Lower subset**: IDPs with a lower status for IDR versus the status of a structural unit with correlation coefficient = 0.856 (Fig 1B–marked as red points).

The remaining points are treated as outstanding points and marked in orange in Fig 1B. Based on two sets of points revealed in Fig 1B, the two linear regression lines were fitted:

*RD*_*IDR*_ = 0.97∙*RD*_*CHAIN*_+0.155 (for green points)
*RD*_*IDR*_ = 0.85∙*RD*_*CHAIN*_+0.0015 (for red points)

The obtained, high values of the slope close to one and very low values of the intercept indicate a high compliance of the statuses of IDR and its unit. It follows from the above equations that the changes of the structure of IDR (i.e. a hydrophobicity distribution measured by the parameter *RD*) in relation to the structure of its structural unit can only take place in the strictly defined ranges defined by the above dependencies. This conclusion of course applies only to the IDPs for which the structure of their IDRs has been solved.

The interpretation of the above presented segmentation suggests the mechanism of using IDRs as a way of determining their biological function. The FOD model reveals the residues involved in ligand binding as those representing a level of hydrophobicity that is locally lower than expected. This applies to the residues present in the cavity prepared for ligand complexation [45]. Similarly, residues showing an excess of hydrophobicity locally on the protein surface are used as the site of complexation of another protein molecule [46].

Our proposed alternative classification of IDRs is based on the following two criteria:

- the values of the parameters *RD* for IDR and its structural unit (chain/domain),
- their relationship to each other.

As it is mentioned in Materials and Methods, the values of parameter *RD* below the cut-off value 0.5 indicate the presence of a hydrophobic core. The values of parameter *RD* are considered as low/high depending on whether they are less / greater than the cut-off value 0.5 (in Fig 1A the perpendicular red lines corresponding to these cut-off values are presented).

Based on the above mentioned two criteria, the collective set of the analyzed IDRs (from S1 Table in S1 Appendix) shown in Fig 1A can be divided into four different groups

1. **Group 1 (FOD-ordered)**: similar, low values of *RD*, both *RD*<0.5 (left-lower quadrant in Fig 1A).
2. **Group 2 (FOD-unordered)**: similar, high values of *RD*, both *RD*>0.5 (right-upper quadrant in Fig 1A).
3. **Group 3 (FOD-unordered)**: different values of *RD*, *RD*<0.5 for the structural unit, *RD*>0.5 for IDR (left-upper quadrant in Fig 1A).
4. **Group 4 (FOD-ordered)**: different values of *RD*, *RD*>0.5 for the structural unit, *RD*<0.5 for IDR (right-lower quadrant in Fig 1A).

According to the FOD-M model, the second parameter *K* expresses the “force” with which the external force field modifies the structure of the hydrophobic core in relation to the system obtained under the sole influence of the water environment. In some cases, such a factor may be an effect of the presence of a disulfide bond, which imposes a structuring different from that preferred by the influence of the water environment. The segmentation of the analyzed IDRs according to the values of parameter *K* are presented in Table 1.

**Table 1:** The segmentation of the analyzed proteins according to the values of parameter *K* (divided into 3 ranges) of IDR and structural unit: The number of proteins in each of 9 groups is presented (in parentheses—the number of proteins with disulfide bonds).

		IDR		
		0≤*K*≤0.5	0.5<*K*<1.5	*K*≥1.5
**structural unit**	0≤*K*≤0.5	42 (5)	23 (5)	9 (2)
	0.5<*K*<1.5	16 (1)	13 (4)	9 (1)
	*K*≥1.5	1	4	3

The proteins defined by the value of parameter *K*≤0.5 are those with the hydrophobicity distribution according to the micelle-like models (3D Gauss distribution). These are proteins with a clearly marked centrally located hydrophobic core. Their folding takes place under the dominant influence of the water environment directing the folding process towards the formation of a centrally located concentration of hydrophobicity. The highest abundance in the group of low values of *K* for both structural unit and IDR proves the presence of many IDRs matching the general molecular form of the hydrophobic core (in chain / domain).

The category of proteins with parameter *K* in the range 0.5<*K*<1.5 are the proteins that are influenced by external factors other than water. Such values are observed for the proteins in contact with the membrane or other factor affecting the non-centric hydrophobicity concentration.

Finally, the proteins having the values of parameter *K*≥1.5 are significantly influenced by external factors. The values of *K* in this range are typical for transmembrane proteins (significantly influenced by the hydrophobic environment of the membrane). It also means structuring resulting from the presence of other (not necessarily membranes) external factors.

The following proteins (marked as 1–4 in Fig 1A) representing the extreme situations in the above mentioned four groups were selected for the detailed analysis:

- 5IXF (the highest accordance as to the very low *RD* values) marked as 1;
- 1OQY (the highest accordance for high *RD* values) marked as 2;
- 2UP1 (the highest discordance for *RD*_*IDR*_≪*RD*_*CHAIN*_) marked as 3;
- 1FHT marked as 4 representing the status *RD*_*CHAIN*_≪*RD*_*IDR*_.

The results of the conducted analysis on the reasons for the observed relationship of the statuses of IDR and SU will be presented in the next four sections.

### 2.2. Group 1

**Fig 2:**
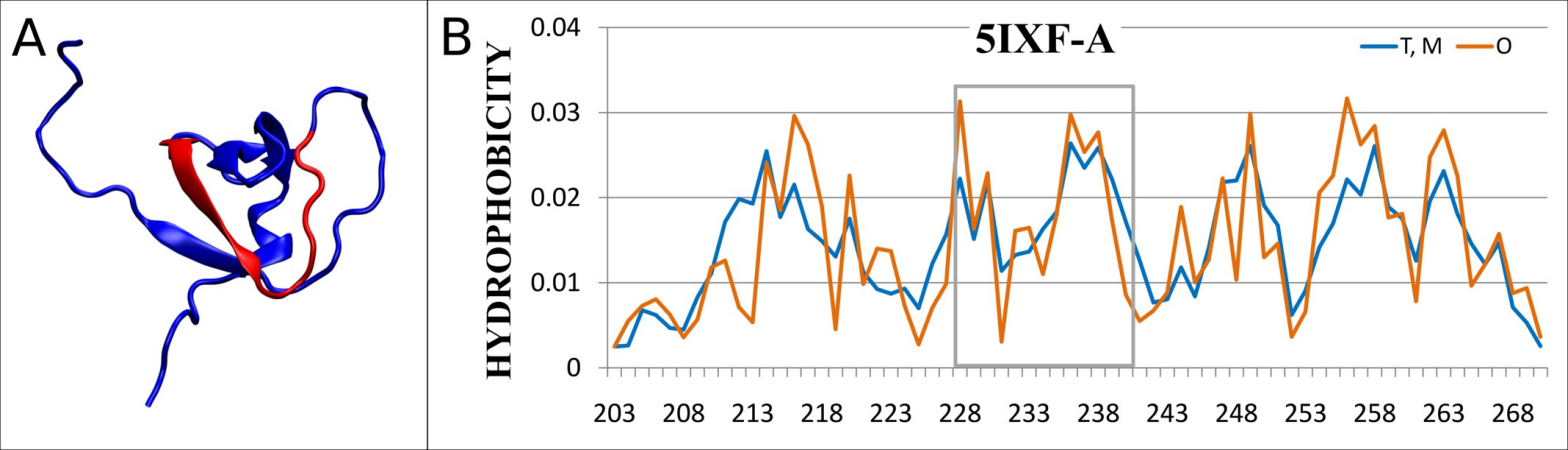
Characteristics of the 5IXF-A. A– 3D structure with highlighted IDR (in red). B–*T* and *O* hydrophobicity distributions (IDR marked by a box).

In this group the status of IDR is similar to the status of the structural unit and the value of *RD* is low. The sample protein representing the status with extremely low value of *RD* for both the structural unit (chain/domain–*RD* = 0.273) and the IDR (*RD* = 0.296) is the signal transducing adapter molecule 2 (PDB ID 5IXF)–denoted as point 1 in Fig 1A. The fragment classified as IDR is a loop with a partially beta-structured form (Fig 2A).

Fig 2B presents the high adaptation (i.e. closeness) of the observed hydrophobicity distribution *O* to the theoretical *T*. It turns out that the lowest value of *D*_*KL*_(*O*|*M*) is obtained for the value *K* = 0 for this protein, so the distribution *M* coincides in this case with the distribution *T*.

The status of this IDR reveals a very high agreement with the micelle-like distribution. From the point of view of the structure of hydrophobic core, this IDR turns out to be an ordered fragment–compatible with the structure of the hydrophobic core covering the entire structural unit. Thus, we introduce a new notation in our classification based on the order of the hydrophobic core as calculated in FOD model–we say this IDR is *FOD-ordered*. This is the example of the evaluation of IDR different from the one existing in the Disprot database.

### 2.3. Group 2

**Fig 3:**
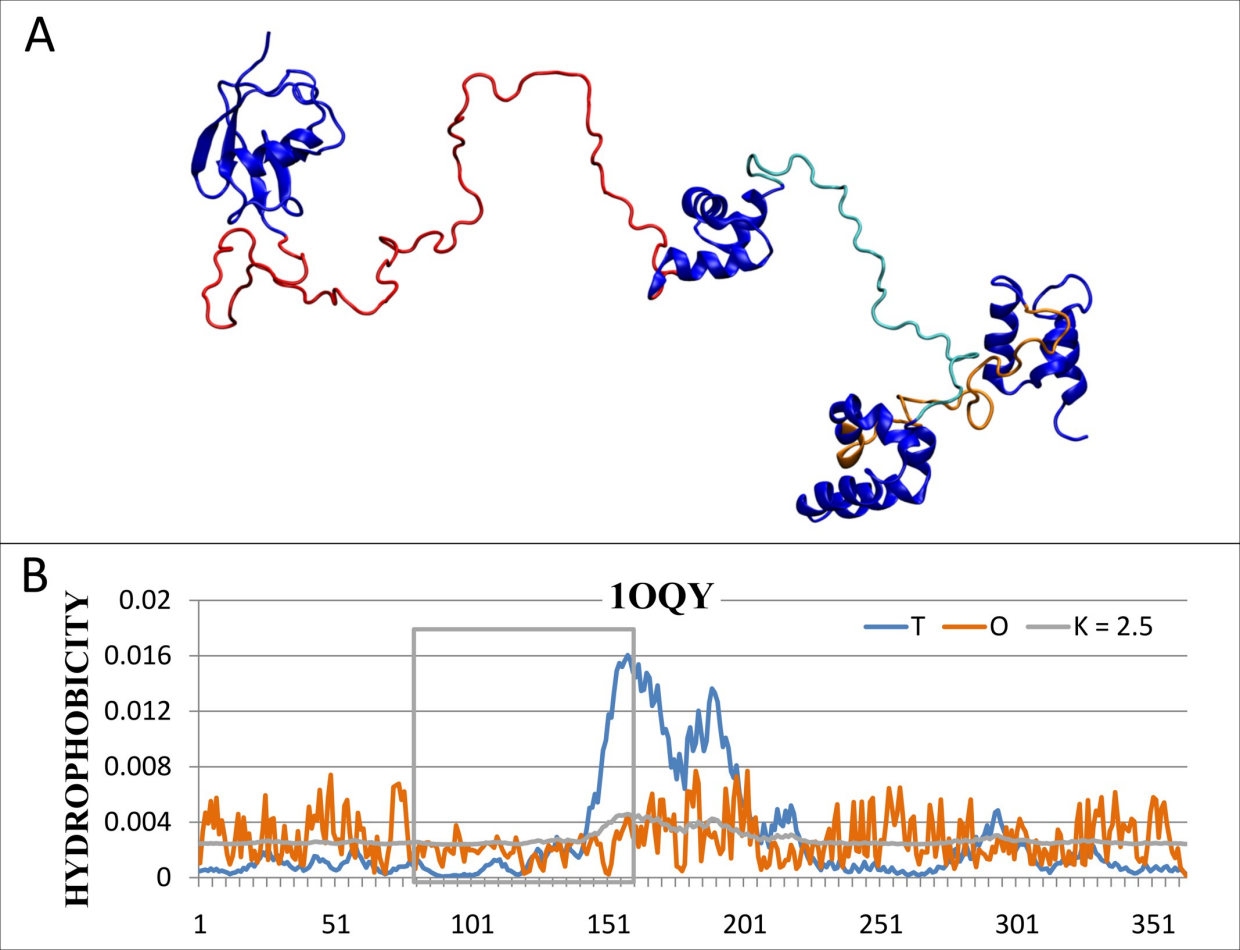
DNA repair protein hHR23a (PDB ID 1OQY). A– 3D structure with IDR (marked as red. B–the distributions *T*, *O* and *M* for *K* = 2.5 (marked in blue, orange and gray, respectively), IDR denoted by a box.

An example of an extremely incompatible system is the DNA repair protein hHR23a (PDB ID 1OQY) (point 2—Fig 1A). This protein is structured in a loose system with three domains located in different parts of the chain. The fragments connecting these domains present a high degree of disorder (in Fig 3A–the analyzed IDR (79–160) marked in red). The structure is perfectly well prepared for the biological function in form of stimulating nucleotide excision repair requiring the adaptation to DNA molecule.

This molecule shows high values of the parameter *RD* for both the structural unit and the IDR (0.829 and 0.910 respectively). From the point of view of the structure of the hydrophobic core, this IDR turns out to be an *unordered* fragment–compatible with the similar unordered structure of its structural unit. The (optimal) values of parameter *K* are the same for the structural unit and IDR and are equal to *K* = 2.5. The distributions *T*, *O* and *M* are presented in Fig 3B (the IDR marked with a box).

The high value of parameter *K* = 2.5 both in the assessment of the entire structural unit and IDR means that a significant environmental factor is required. This factor turns out to be the proteasomal subunit S5a with which the discussed protein interacts. Based on the interpretation according to the FOD-M model—the structure represented by the discussed protein cannot exist in the water environment. The value of *K* = 2.5 can also be an assessment of the strength of external field modification which for the discussed protein is the proteasomal subunit S5a.

According to our classification based on the order of the hydrophobic core as calculated in FOD model–we say this IDR is *FOD-unordered*. This is the example of the evaluation of IDR “consistent” with that existing in the Disprot database.

### 2.4. Group 3

**Fig 4:**
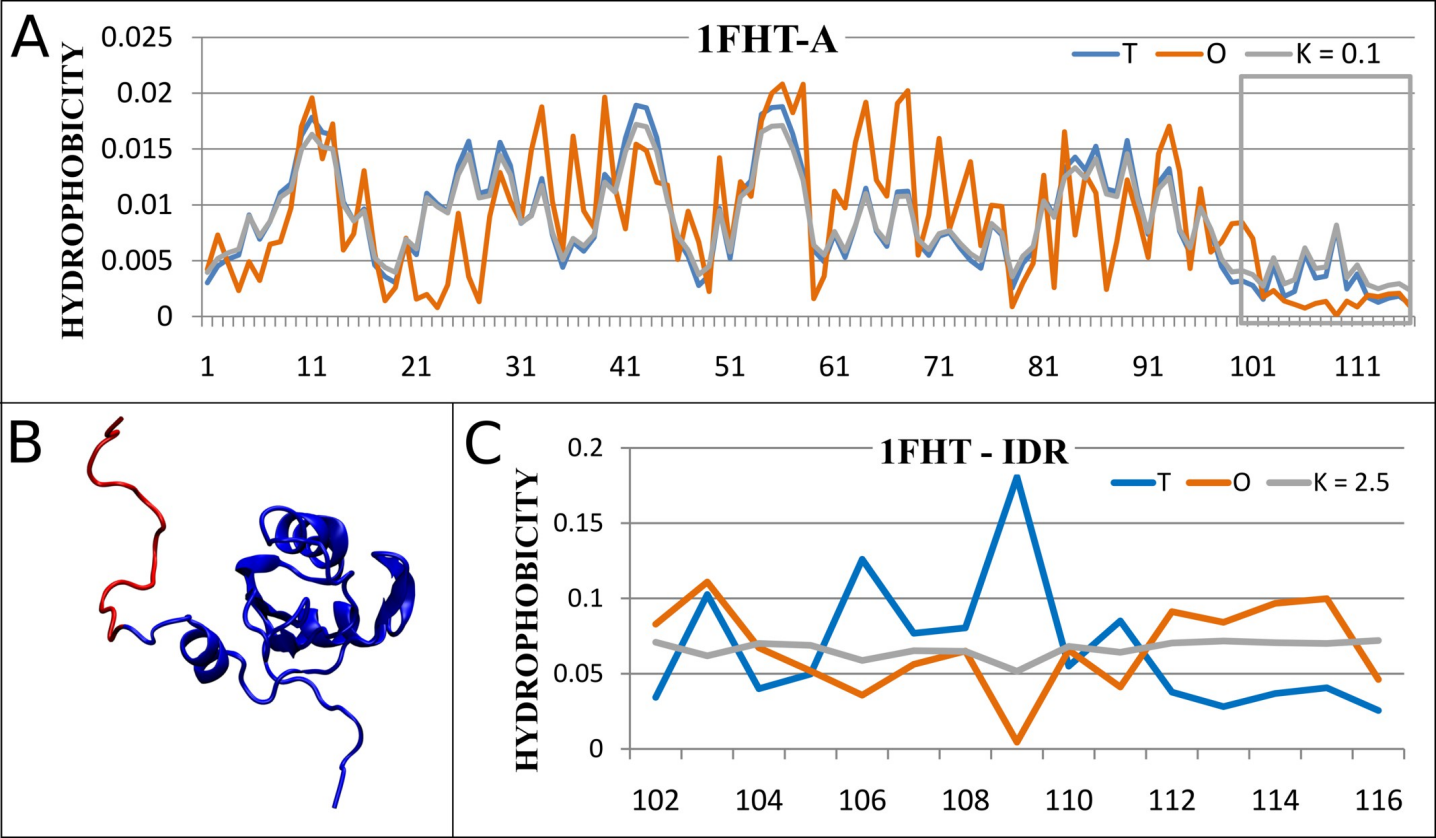
RNA-binding domain of the U1A spliceosomal protein (PDB ID 1FHT). A—the distributions *T*, *O* and *M* (corresponding to optimal *K* = 0.1) in whole structural unit (marked in blue, orange and gray respectively), IDR denoted by a box; B– 3D structure with IDR marked in red; C–the distributions *T*, *O* and *M* (corresponding to the optimal *K* = 2.5) in IDR.

An example of a protein with the status of IDR greater than 0.5 and significantly different from that for the entire structural unit is RNA-binding domain of the U1A spliceosomal protein U1A117 (PDB ID 1FHT) (point 3—Fig 1A). The value of parameter *RD* for a structural unit is equal to 0.360 and the optimal value of parameter *K* = 0.1 (the value corresponding to the smallest *D*_*KL*_(*O*|*M*)). The status of IDR (terminal segment 100–116 –see Fig 4) is expressed by the values *RD* = 0.790 and optimal *K* = 2.5.

This means that a whole chain represents a status consistent with a micelle-like distribution (despite the absence of order in IDR). The term micelle-like expresses the presence of a concentration of hydrophobicity in the central part (a core) of a protein with a hydrophilic surface.

According to our classification based on the order of the hydrophobic core as calculated in FOD model–we say this IDR is *FOD-unordered*. This is the second example of the evaluation of IDR “consistent” with that existing in the Disprot database.

### 2.5. Group 4

**Fig 5:**
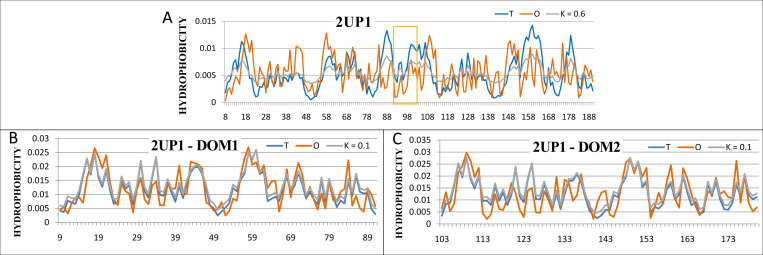
The distributions *T*, *O*, *M* for two-RRM domains of hnRNPA1 (PDB ID - 2UP1). A–a whole chain treated as a structural unit for the FOD calculation; segment IDR is marked by a box); B. C–the two domains treated as independent structural units.

The protein two-RRM domain of hnRNP A1—PDB ID 2UP1 (point 4 in Fig 1A) is the sample one having different statuses for a structural unit and its IDR. The whole structural unit presents a relatively high value of a parameter *RD* = 0.571 (the optimal value of *K* = 0.6) while its IDR–very low value *RD* = 0.214 (the optimal *K* = 0.0). The plots of distributions *T*, *O* and *M* (corresponding to the optimal value of parameter *K*) reveal the reason for the high value of unit’s parameter *RD*–the mismatch between the observed distribution *O* and the theoretical *T* (see Fig 5A).

**Fig 6:**
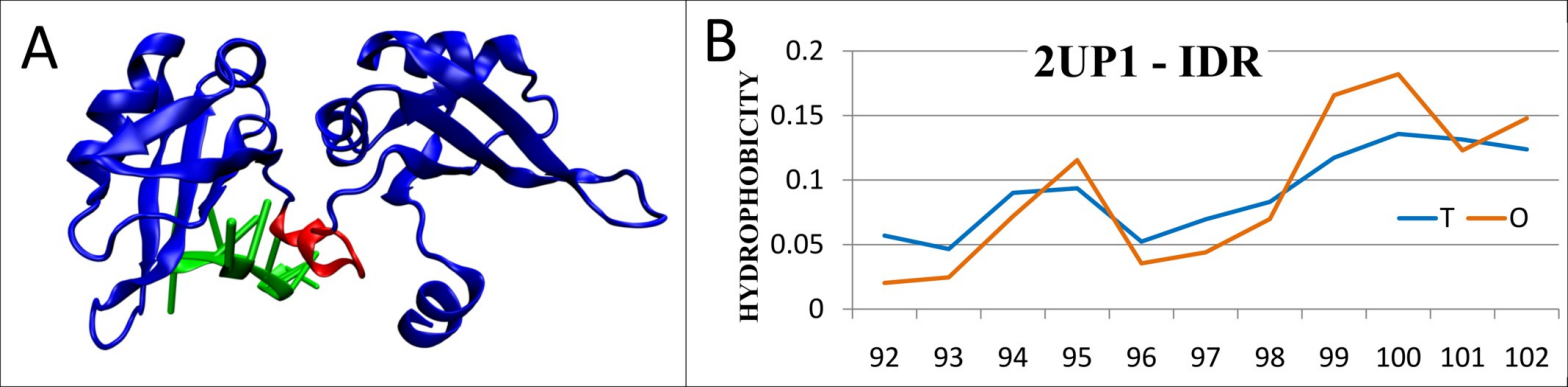
Characteristics of two-RRM domain of hnRNP A1 (PDB ID: 2UP1). A– 3D structure with highlighted IDR (red) and marked single-chain DNA fragment complexed by this protein (green); B–the distributions *T* and *O* for the optimal *K* = 0.

The analysis of the type of divergence of the *O* distribution from the *T* one suggests the presence of a cavity revealed in the form of a hydrophobicity deficit (sections: 82–90, 98–106. 152–168) (Fig 5A). The cavity is present between the two domains, each of which separately exhibits a high degree of ordering consistent with the micelle-like form (Fig 5B and 5C). The identified IDR is located between the domains, creating a specific linker between them that plays an essential role. The presence of a cavity is associated with the possibility of DNA strand complexation which requires the flexibility and movement of the domains (Fig 6A). Nevertheless, in the structure obtained by crystallization, the IDR is located in the optimal position from the point of view of the hydrophobic core structure in the local range.

The status of this IDR reveals a very high agreement (Fig 6B) with the micelle-like distribution being an *ordered* fragment (according to the FOD model)–but incompatible with the entire structural unit. We say this IDR is *FOD-ordered*. This is the second example of the evaluation of IDR different from the one existing in the Disprot database.

### 2.6. The role of disulfide bonds

It is widely known that disulfide bonds and hydrophobic core are regarded as factors stabilizing the tertiary structure. The status of the fragments of protein chain involved in the construction of the hydrophobic core and covered by disulfide bonds is discussed in [47]. There are examples of proteins in which the disulfide bonds favor the ordered hydrophobic core, but also the opposite ones where the local maladjustment to the micelle-like system is imposed.

From the discussed set of IDPs, three proteins were selected to investigate the status of chain’s fragments as well as the fragments indicated in the Disprot database as IDRs both covered by disulfide bonds (the status was measured by the value of parameters *RD* and *K* form FOD-M model). These are Pleiotrophin—heparin binding protein (PDB ID - 2N6F), Vascular endothelial growth factor B (PDB ID - 2VWE) and Glycoprotein hormones alpha chain (PDB ID - 1DZ7. Table 2 shows the calculated status of the chain’s fragments in the mentioned proteins.

**Table 2:** The values of parameters *RD* and *K* for the chain’s fragments covered by the disulfide bonds (SS). The fragments marked with stars–are indicated in the DisProt database as IDRs. Numbering in accordance with RCSB database; (r): Reference protein.

PDB ID	fragment	*RD*	*K*
2N6F	15–44	0.754	1.1
	23–53	0.719	1.5
	30–57	0.731	1.5
	67–99	0.560	0.6
	77–109	0.624	1.2
	0–14*	**0.628**	**2.5**
	52–63*	**0.503**	**0.2**
	108–136*	**0.572**	**0.6**
2VWE	26–68	0.608	0.5
	57–101	0.557	0.5
	61–103	0.586	0.6
	16–25*	**0.780**	**0.8**
1DZ7	7–31	0.665	0.5
	10–60	0.437	0.3
	28–82	0.439	0.3
	32–84	0.441	0.3
	59–87	0.616	0.4
	9–33*	**0.656**	**0.5**
2L42 (r)	1–97 –whole chain	0.387	0.0

In order to assess the influence of disulfide bonds in IDPs, the reference protein–the DNA-binding protein RAP1 from *Saccharomyces cerevisiae* (PDB ID - 2L42) was also selected, almost entirely having the IDR status, in which there are no disulfide bonds.

**Fig 7:**
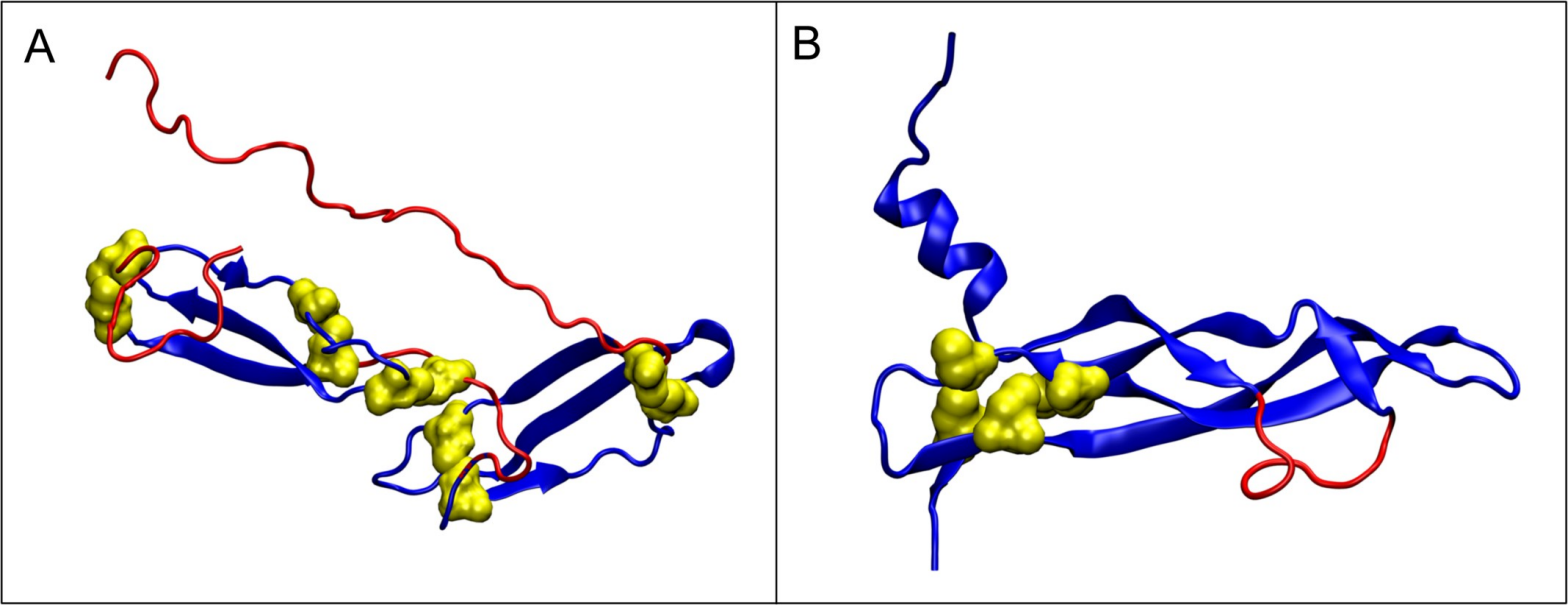
3D presentation of the selected proteins with disulfide bonds present. A - 2N6F. B - 2VWE. Disulfide bonds—yellow space filling. IDRs—red.

**Fig 8:**
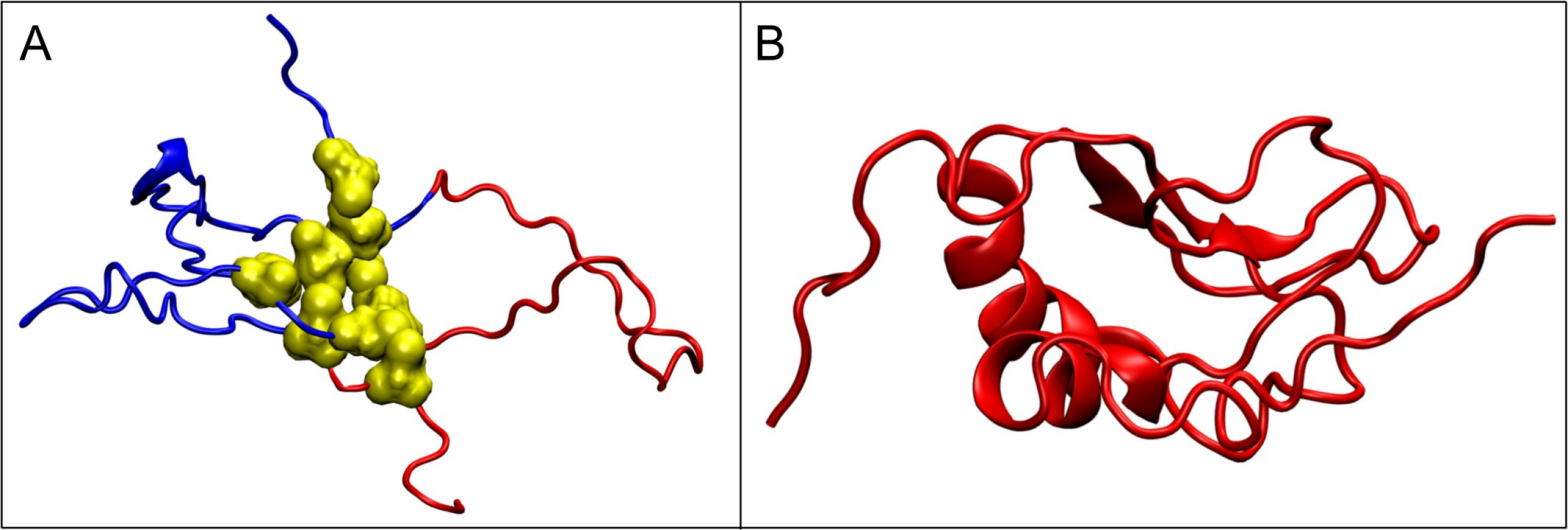
3D presentation. A– 1DZ7 (yellow–Cys residues in disulfide bonds). B– 2L42 (red fragments–classified in DisProt as IDR).

The Figs 7 and 8 present 3D structure of the mentioned proteins with marked IDR.

In the case of 2N6F and 2VWE proteins, the status of all segments covered by the disulfide bonds (including those being IDRs according Disprot database) present a maladjustment to the hydrophobicity ordering according to the micelle-like distribution—the observed hydrophobicity distribution *O* is inconsistent with the theoretical distribution *T* (*RD*>0.5). Also in the case of 1DZ7 protein, the fragment 9–33 being IDR and covered by the disulfide bonds, shows a similar maladjustment. The IDRs present in these fragments are *FOD-unordered* according to our classification. A specific disorder of the hydrophobicity distribution in these fragments is a form of encoding the possibility of interaction with other molecules. It can be speculated that the FOD-unordered status (disordered according to the Disprot database) of these IDR fragments is the “result” of the structure stiffened by the disulfide bonds (i.e. the constraints introduced by them) to enable performing a specific function. In these cases, the structure is stabilized by the disulfide bridges.

**Fig 9:**
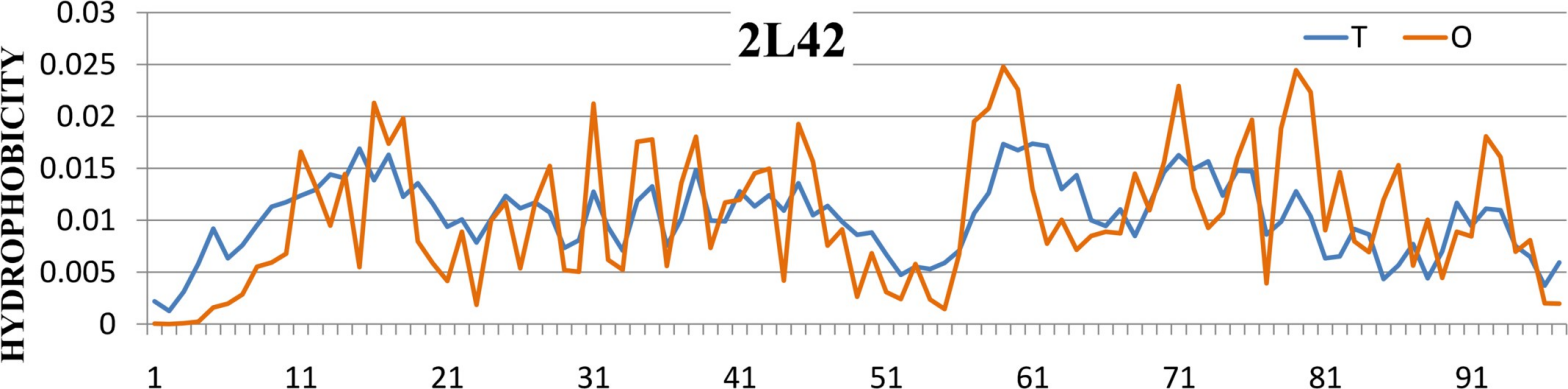
The *T* and *O* distributions for 2L42 protein (profile *M* for *K* = 0.0 coincides with profile *T*).

The 2L42 protein, almost entirely scored as IDR, presents the perfect adjustment of the observed hydrophobicity distribution *O* to the theoretical distribution *T* (see Fig 9). According to our classification this protein is FOD-ordered. Here, the perfect hydrophobic core is a factor stabilizing the structure (disulfide bonds not present) in water (as *K* = 0.0). Such a core is the result of the action of an external force field expressed by means of a 3D Gaussian function.

An ordered hydrophobic core is present as long as the outer field remains in this form. If the external conditions change, the structure of these proteins will probably change as it adapts to the new conditions. The 2L42 protein (having no disulfide bonds) may change the structure globally adapting to changed conditions. The degree of deformation probably depends on the degree of change in the characteristics of the external field (expressed in the FOD-M model by the growing value of parameter *K*). Returning to the conditions of the aquatic environment enables the reconstruction of a perfect hydrophobic core. These changes are possible due to the relatively low presence of secondary structures in 2L42.

## 3. Discussion

The activity of proteins is inseparably related to the water environment. Protein folding—a largely spontaneous process—takes place with the active participation of the surrounding water. It is a specific solvent of a polar nature that directs the folding process towards the isolation of hydrophobic residues in the center of the molecule due to simultaneous exposure of polar residues on the surface. The result of this process is the formation of globular molecules with a hydrophobic core present in the center. However, the ordering of hydrophobicity in proteins is achieved to varying degrees. The determined inability to create a micelle-like system with full ordering according to the 3D Gauss function (i.e. as in FOD-M model) is encoded in an amino acid sequence. The local disagreement of the centric order is an element that carries information about biological activity, the local hydrophobicity deficit is in most cases related to the presence of ligand-binding cavity (substrate) [45]. The local excess of hydrophobicity on the surface of a molecule is a form of encoding the complexation site of another protein [46]. Therefore, the protein can be defined as "intelligent micelle", in which the region of mismatch with the micellar distribution carries information about its specificity. A perfect micelle is deprived of any form of specificity. The form and degree of discordance in micelle-like structure in protein endows the protein with specificity.

In the FOD-M model used in the current analysis—the fit to the environment is expressed by the value of parameter *RD*, while the strength with which other factors shape the structure of the protein (or its fragments) is determined by the value of parameter *K*. The parameter *K* can be used as a measure of a mismatch between the given protein (or a section of a polypeptide chain) and the micelle-like system, regardless of the factors causing it. Moreover, the parameter *K* can also be used to assess the degree of the interference of a factor different from the water environment. Such interaction–especially in the membrane environment, is treated as conditioning biological activity [48–50].

The high variability and diversity of IDPs forms assessed on the proteomic scale (three superkingdoms) correlates with the degree of complexity of the system (not without exceptions) and it also turns out to be variable from the point of view of clade organization and it is associated with clade-specific functions [51]. This observation may be related to the revealed large variability in the coherence of the organization of the structural unit and IDR: from highly ordered to completely disordered taking into account the structure of the hydrophobic core as the evaluation criterion. The results presented here seem to be coherent with the observations reported in the domain and extra-domain IDR assessment [52,53].

In the context of IDPs, an important example is the protein discussed in this paper: DNA-binding protein rap1 (PDB ID 2L42) and its relation to (PDB ID - 1DZ7). With a very low proportion of the secondary structure (less than 30% of the chain length) and in the absence of disulfide bonds. This protein turns out to be stabilized mainly by the hydrophobic core. The status of this protein described in this way (*K* = 0) seems to be largely dependent on and sensitive to changes in the characteristics of the environment. As long as the outer polar force field (environment) retains its characteristics. The protein retains its highly ordered structure based on the present hydrophobic core. Each—probably even minimal—change in the characteristics of the external field causes easy adaptation to the new conditions of this protein. This feature is expected in the preparation of a protein for interaction in this case of DNA complexation.

The analysis of the structuring of the hydrophobicity distribution—the hydrophobic core—does not distinguish the IDRs as different from the structural unit, constituting an integral part of the hydrophobicity distribution consistent with that present in the structural unit (chain / domain). The low presence of secondary structure does not mean that the hydrophobicity system does not match the status of a structural unit (chain / domain). The presence of an ordered core, in turn, favors the stabilization of the tertiary structure. If, however, it results only from the presence of an ordered core—then according to the fuzzy oil drop model—it means a high dependence on the environment. In such a case, the change in environmental conditions (measured by the value of the parameter *K*) may have a quite radical effect on the change in protein structuring, which is the result of targeting coming from the environment.

The investigated set of IDPs presents the linear relationship between the hydrophobicity distributions(measured by the parameter *RD*) of IDR and its SU. This interesting fact has been analyzed in detail in this paper. It follows that the structural changes of IDR (measured by the parameter *RD*) can only take place in the strictly defined ranges defined by the linear dependencies.

The proposed alternative classification of IDRs based on the level of ordering of hydrophobic core (measured in FOD-M model by the parameter *RD*) complements the existing classification of IDPs according to the Disprot database resulting in FOD-ordered or FOD unordered IDRs. The presence of a hydrophobic core in IDPs/IDRs sheds new light on the assessment of the stabilization of these proteins. These obtained results of course apply only to the IDPs for which the structure of their IDRs has been solved.

In this paper we describe the revealed phenomenon–the near linear dependency between hydrophobicity distribution measured by the parameter of the FOD-M model using the examples of the selected IDPs. This is the first stage of our in silico experiment, shedding new light on the IDPs through the prism of the FOD-M model. To try to answer the question why is it like this—a whole series of new research is needed, which will be the topic of our further work.

The inclusion of proteins derived from *Mus Musculus* reduces the strength of the dependence between parameters RD for IDR segments and the entire structural unit. Another conclusion from the analysis of *Mus Musculus* proteins is the new idea of expansion the FOD-M model with a different chaotropic factor.

It should be noted that the currently discussed proteins are those for which the structure of the IDR segment is available (in PDB). Probably those examples of proteins, where the structure of IDR segments is not experimentally recognizable, would change the assessment of the discussed state of affairs.

The team plans to analyze the proteins containing IDR segments that do not have an experimentally determined structure by using their structure predicted by AlphaFold package. Such research will significantly increase the size of the database and will probably bring a new look at the problem discussed here.

## 4. Materials and methods

### 4.1. Data

The set of analyzed proteins is derived from the *Homo Sapiens* proteins present in the DisProt database [20–24] (721—as accessed Apr 2021). The fuzzy oil drop model used for the analysis requires the 3D structure of a protein under study. Additionally, in order to assess the status of IDR, it is also necessary to know its structure. Therefore, from the 721proteins of *Homo Sapiens* present in the DisProt database—the structures available in PDB limit this number to 454. Moreover, the known solved structure of IDRs is available only for 75 proteins. The final list of proteins analyzed in the present work is given in S2 Table in S1 Appendix.

It should be clearly emphasized that the analyzed proteins are available in the PDB database. IDPs are very often available without any specific IDRs structure. Therefore, the conclusions proposed here should be limited to a special group of IDPs, where the flexibility of the structure is so low that it is possible to solve the structure of their IDRs.

The below described FOD-M model was used to analyze these proteins classified as IDPs according to the DisProt database (S1 Table in S1 Appendix). The definition of a domain is taken from PDBsum database [54].

### 4.2. Description of the FOD-M model

The Fuzzy Oil Drop (FOD) model has already been described many times in the literature, see for example [42–44]. The FOD model assumes that a polypeptide chain is composed of amino acids that exhibit the nature of bi-polar molecules that in the aquatic environment tend to generate a micelle-like structure with a centric hydrophobic core. This *idealized (theoretical) distribution T* can be modeled by a 3D Gaussian function on the protein body. The sequence limitations where the amino acids are joined by the covalent bonds results in the *observed distribution O* matching the theoretical one to a greater or lesser degree. Let us formally define the two distributions *T* and *O*.

The theoretical distribution *T* is defined by the hydrophobicity $H_{i}^{T}$ (*i* = 1,…,*N*, *N* being the number of residues) expressed by the value of 3D Gaussian function at position of *i*-th effective atom (i.e. the average position of atoms that make up the *i*-th residue):

$$H_{i}^{T}=\frac{1}{H_{sum}^{T}}exp\left(\frac{-{\left(x_{i}-\bar{x}\right)}^{2}}{2\sigma_{x}^{2}}\right)exp\left(\frac{-{\left(y_{i}-\bar{y}\right)}^{2}}{2\sigma_{y}^{2}}\right)exp\left(\frac{-{\left(z_{i}-\bar{z}\right)}^{2}}{2\sigma_{z}^{2}}\right)$$

The values for the *σ*_*x*_, *σ*_*y*_, *σ*_*z*_ parameters are determined based on the molecule under consideration.

The observed distribution *O* is defined by the hydrophobicity $H_{i}^{O}$ at the position of the *i*-th effective atom according to the Levitt [55]:

$$H_{i}^{O}=\frac{1}{H_{sum}^{O}}\sum_{j}\{\begin{array}{c} (H_{i}^{r}+H_{j}^{r})\left(1-\frac{1}{2}(7{\left(\frac{r_{ij}}{c}\right)}^{2}-9{\left(\frac{r_{ij}}{c}\right)}^{4}+5{\left(\frac{r_{ij}}{c}\right)}^{6}-{\left(\frac{r_{ij}}{c}\right)}^{9})\right),\;\mathrm{for}\;r_{ij}\leq c\\ 0,\;\mathrm{for}\;r_{ij}>c \end{array}$$

The hydrophobicity $H_{i}^{O}$ collects the hydrophobic interactions in distance-dependent form as given in the above formula with the cutoff distance (*c*) according to the original work [55] - 9Å. The $H_{i}^{r}$ and $H_{j}^{r}$ denote the intrinsic hydrophobicity of *i-th* and *j-th* residues. The purpose of denominators $H_{sum}^{T}$ and $H_{sum}^{O}$—being the sum of all $H_{i}^{T}$ and $H_{i}^{O}$ respectively, is to normalize the hydrophobicities to the range 〈0,1〉.

**Fig 10:**
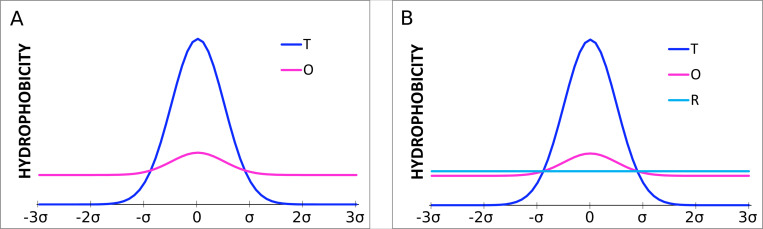
Examples of distributions. A–distribution *O* (pink) and reference Gaussian distribution *T* (with parameter *σ*) expressing the presence of the central hydrophobic core (dark blue). B–the second reference distribution *R* (light blue) superimposed—expressing the lacking of any variation in the hydrophobicity.

The example of the theoretical *T* (dark blue) and observed *O* (pink) hydrophobicity distribution is presented in Fig 10A.

The *T* and *O* distributions can be quantitatively compared using the *divergence entropy D*_*KL*_ between the two distributions *P* and *Q* introduced by Kullback-Leibler [56].

$$D_{KL}(P|Q)=\sum_{i=1}^{N}P_{i}\;\log_{2}\frac{P_{i}}{Q_{i}}$$

where *P*_*i*_–probability observed (in our model–$H_{i}^{O}$, the observed hydrophobicity for the *i*-th residue), *Q*_*i*_–reference probability (in our model–$H_{i}^{T}$, the theoretical hydrophobicity for the *i*-th residue).

Next we introduce the *reference distribution R*, being the uniform one where *i*-th residue is assigned the same hydrophobicity *R*_*i*_ = 1/*N*, *N* being the number of residues in a polypeptide chain (Fig 10B–light blue line). This distribution represents a lack of any variation in the hydrophobicity within a molecule.

A comparison of two *D*_*KL*_ values, *D*_*KL*_(*O*|*T*) and *D*_*KL*_(*O*|*R*) shows which “distance” is closer. The values *D*_*KL*_(*O*|*T*) less than *D*_*KL*_(*O*|*R*) allow inferring the presence of a centric concentration of hydrophobicity and thus the presence of a hydrophobic core.

To eliminate the necessity of using the two values, the following parameter *RD*—*Relative Distance* is introduced:

$$RD=\frac{D_{KL}(O|T)}{D_{KL}(O|T)+D_{KL}(O|R)}$$

The parameter *RD* expresses the degree of adjustment of the hydrophobicity distribution observed in a given structure—resulting from the distribution of residues with a specific intrinsic hydrophobicity to the idealized distribution expressed by a 3D Gaussian function spread over the folding chain at a given moment of the folding process.

The values of *RD*<**0.5** (being the *cut-off value*) indicate the presence of the hydrophobic core generated during the folding process. The ideal theoretical hydrophobicity distribution in the protein means the micelle-like state guaranteeing solubility without the possibility of interaction except for random interaction with ions or low molecular weight compounds. The larger deviations of the *O* from the *T* hydrophobicity distribution (i.e. when the cut-off value 0.5 is exceeded) carry information about the *specificity* of a given protein, enabling, for example, interaction with a specific ligand by the appropriate adjustment of the interaction field. Of course, it is also possible to bind the polar ligand on the protein surface without disturbing the structure of the hydrophobic core.

The modification of the FOD model, the so-called FOD-M model [42], extending the participation of a non-polar environment in protein folding relies on introducing the structural specificity of membrane proteins—including membrane proteins serving as an ion channel [44].

Following the hydrophobicity distribution in membrane proteins (where an exposure of hydrophobic residues is expected on the surface and the presence of polar ones—in the center), we define the *modified hydrophobicity distribution M* which is “inverted” to the centric theoretical distribution *T* and can be expressed by the function:

$$M_{i}=T_{MAX}-T_{i}$$

where *T*_*MAX*_ is the maximum value in the theoretical distribution *T*.

The distribution *T* is modified, assigning to individual residues a status in the form of complement to the value expected for the centric distribution. However, it turns out that the omnipresence of the aquatic environment also imprints the structure of the membrane protein. Therefore, the external field directing the protein folding process turns out to be a consensus between the centric field and the inverted one, and can be expressed as:

$$M_{i}={\left[T_{i}+{\left(T_{MAX}-T_{i}\right)}_{n}\right]}_{n}$$

where the index *n* denotes normalization which relies on dividing each element (i.e. the partial hydrophobicity from *i*-th residue) of the set by the sum of all elements in it. After normalization, the sum of all elements is equal to 1.

The *M* distribution expresses the influence of the membrane environment in the extreme case, which is the membrane, being the fully hydrophobic environment. The coefficient *K* was additionally introduced to make the definition of a distribution *M* more universal:

$$M_{i}={\left[T_{i}+{K(T_{MAX}-T_{i})}_{n}\right]}_{n}$$

The coefficient *K* expresses the consensus between the water environment (centric hydrophobic core) and the hydrophobic environment of the membrane (or presence of any hydrophobic compound modifying the idealized distribution expressed by 3D Gauss function). Values of the coefficient *K* close to 0 represent proteins with a high degree of centric hydrophobicity while those close to 1—represents structures with a significant part of a membrane environment. It also turns out that the value of a parameter *RD* is highly correlated with the value of coefficient *K*. Both these values express the degree of deviation from the micelle-like hydrophobicity distribution within the protein. The value of parameter *RD* represents the difference from the centric distribution while the value of coefficient *K—*measures the participation of other than polar factors influencing the folding process.

**Fig 11:**
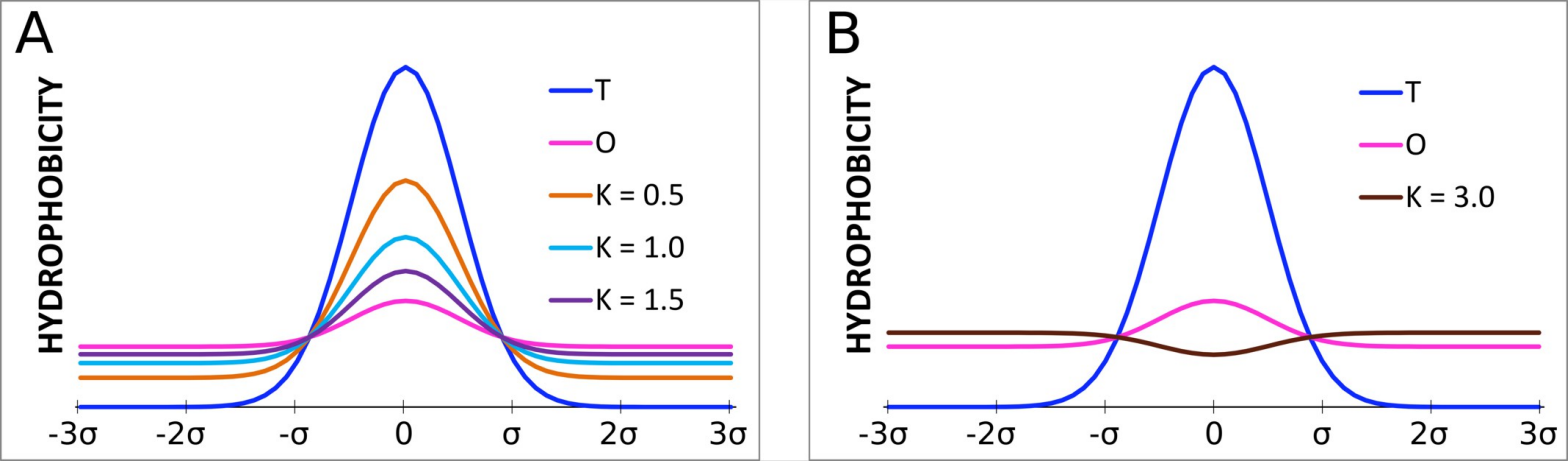
A: The comparison of plots of distributions *M* for the three different values of *K*; B: Plot of distribution *M* for a very high value of *K* (3.0). The distributions *T* and *O* marked in navy blue and pink respectively.

The sample plots of distribution *M* for the three values of coefficient *K* (*K* = 0.5;1.0;1.5) are presented in Fig 11A. Fig 11B shows the plot of distribution *M* with a very high value of *K* (*K* = 3) which completely eliminates the presence of a maximum, introducing a minimum in its place. Such situations are observed for ion channels.

**Fig 12:**
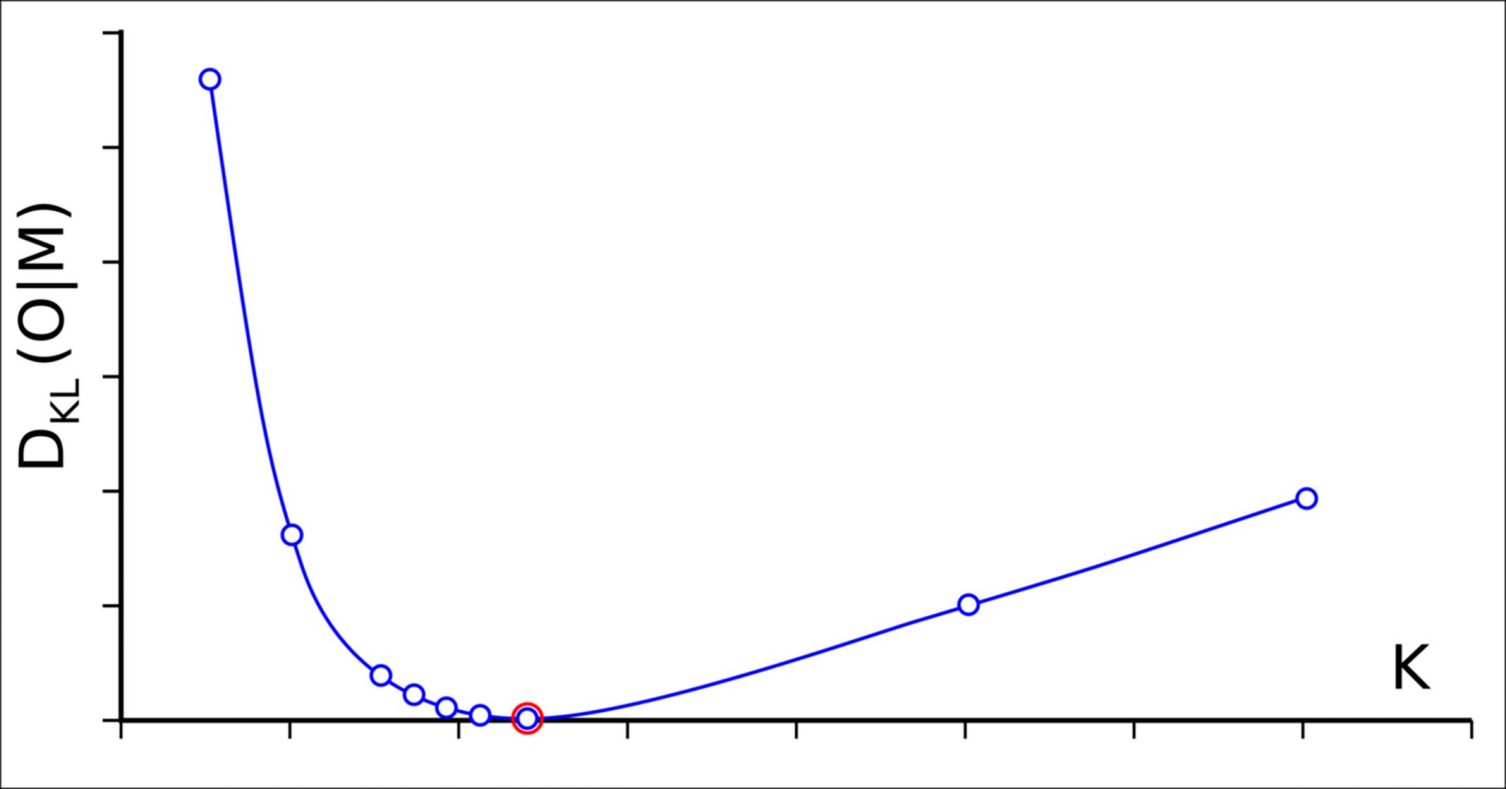
Determining the optimal value of coefficient *K*. The lowest *D*_*KL*_ value is marked (red circle).

Next, the optimal value of coefficient *K* is determined by seeking for the value of *K* corresponding to the smallest value *D*_*KL*_(*O*|*M*) of the distance between the two distributions: observed *O* and membrane *M*. For such optimal value of coefficient *K* (see Fig 12).

The parameter *RD* expressing the relative distance between the distributions *O* and *T* is supplemented with the parameter *RD* calculated for the relative distance between the distributions *O* and *M*_*opt*_ (the distribution *M* corresponding to the optimal value of a coefficient *K* as described above):

$${RD}_{Kopt}=\frac{D_{KL}(O|T)}{D_{KL}(O|T)+D_{KL}(O|M_{opt})}$$

Summarizing, the FOD model considers only the water environment expressing the external force field as a 3D Gauss function, while the FOD-M model allows for the presence of other environmental factors. This model enables the quantification of the factor modifying the polar water environment.

### 4.3. Programs used

The program used for the calculation of the parameter *RD* was implemented in collaboration with the Sano Centre for Computational Medicine (https://sano.science) and is running on resources contributed by ACC Cyfronet AGH (https://www.cyfronet.pl) in the framework of the PL-Grid Infrastructure (https://plgrid.pl). This program can be used freely via a web wrapper available at https://hphob.sano.science.

The VMD program was used to present the 3D structures [57,58].

### 4.4. Calculation procedure

For the purposes of this study, it is necessary to define the term “*structural unit*”. The model used to express the hydrophobicity distribution by the 3D Gaussian function works best for the globular proteins. Therefore, the structural unit on which the mentioned function is spread can be a complex (regardless of the number of chains), a single chain (regardless of the shape taken by the structure of the chain) or a domain—a unit, which is treated as an effect of individual independent folding. In both: the complex structure and in the multi-domain polypeptide chain it is possible to determine the status of the lower-level structural units: a chain in a structure of a complex, a domain in a structure of a chain. In addition, it is also possible to evaluate the status of the selected fragment—including, in particular, the intrinsically disordered region (IDR) as a component of a chain or domain.

The procedure for calculating parameter *RD* (i.e. a status) is as follows: first, the parameters of the 3D Gaussian hydrophobicity distribution are determined based on the spatial structure of the structural unit, that is, the entire chain or domain, if it has been distinguished. Then, while maintaining the same 3D Gaussian distribution parameters:

1. the status of the entire structural unit and
2. the status of the IDR is determined.

The method of calculating the *M* profile described in 4.2 in the calculations for IDR needs to be clarified. Here, the *T*_*MAX*_ value is determined within a given IDR (not the whole structural unit), and then normalizations are performed as described in section 4.2.

## Supporting information

S1 Appendix The results of the analyzed *Homo Sapiens* IDPs based on the FOD-M model. General characteristics of the *Mus Musculus* IDPs based on the FOD-M model. This appendix contains tables: **SI Table 1**. List of *Homo Sapiens* proteins under consideration from DisProt database with the disordered fragments as present in the structure available in PDB; **SI Table 2**. The values of parameter *RD* and of optimal parameter *K* according to the segmentation shown in Fig 1B; **SI Table 3**. List of *Mus Musculus* proteins under consideration with disordered fragments; **SI Table 4**. The values of parameter *RD* and of optimal parameter *K* corresponding to the smallest value *D*_*KL*_(O|M) (calculated in the FOD-M model) for a structural unit (chain/domain) and IDR.
(DOCX)
