# Supplementary material for: New insights into disordered proteins and regions according to the FOD-M model
Source: PLoS One. 2022 Oct 10;17(10):e0275300. doi: 10.1371/journal.pone.0275300 (PMC9550084; doi:10.1371/journal.pone.0275300)
Supplement: S1 Appendix — General characteristics of the Mus Musculus IDPs based on the FOD-M model. This appendix contains tables: SI Table 1. List of Homo Sapiens proteins under consideration from DisProt database with the disordered fragments as present in the structure available in PDB; SI Table 2. The values of parameter RD and of optimal parameter K according to the segmentation shown in Fig 1B; SI Table 3. List of Mus Musculus proteins under consideration with disordered fragments; SI Table 4. The values of parameter RD and of optimal parameter K corresponding to the smallest value DKL(O|M) (calculated in the FOD-M model) for a structural unit (chain/domain) and IDR. (DOCX) [file pone.0275300.s001.docx]

**S1 Appendix**

**The results of analyzed *Homo Sapiens* IDPs based on the FOD-M model**

**S1 Table.** List of *Homo Sapiens* proteins under consideration from DisProt database with the disordered fragments as present in the structure available in PDB. Column DisProt ID gives the identification number according to the DisProt database. D – domain treated as a structural unit – all others – chain is treated as a structural unit.

| PDB ID | DisProt ID  DPxxxxx | Functional characteristics | domain | ref |
| --- | --- | --- | --- | --- |
| 1B3J | 670 | immune system | D | [1] |
| 1D7Q | 903 | gene regulation |  | [2] |
| 1DZ7 | 2922 | glycoprotein |  | [3] |
| 1EOT | 641 | phosphotransferase |  | [4] |
| 1F9X | 1773 | apoptosis inhibitor |  | [5] |
| 1FHT | 1857 | ribonucleoprotein |  | [6] |
| 1LXL | 298 | inhibitor of programmed cell death |  | [7] |
| 1NI2 | 775 | structural protein | D | [8] |
| 1O9U | 385 | glycogen synthase kinase-3 bet | D | [9] |
| 1OQY | 156 | DNA repair protein hhr23a |  | [10] |
| 1OWA | 1700 | erythrocyte alpha spectrin |  | [11] |
| 1P22 | 1119 | signaling protein |  | [12] |
| 1PC2 | 457 | mitochondria fission protein fis1 –  unknown function |  | [13] |
| 1R4M | 2302/2306 | e1-ubiquitin-like protein |  | [14] |
| 1R8U | 356 | transcription/transcription activa |  | [15] |
| 1RXR | 62 | transcription factor |  | [16] |
| 1SOH | 1883 | lipid transport |  | [17] |
| 1TXI | 184 | gene regulation |  | [18] |
| 1UAD | 581 | enndocytosis/exocytosis |  | [19] |
| 1UEC | 2254 | signaling protein |  | [20] |
| 1XPA | 243 | RNA repair |  | [21] |
| 1Z1M | 334 | ligase |  | [22] |
| 1ZZA | 162 | membrane protein |  | [23] |
| 2BID | 1662 | pro-apoptotic protein bid |  | [24] |
| 2D3V | 2812 | Immune system | D | [25] |
| 2D5R | 794 | transcription |  | [26] |
| 2EYW | 748 | signaling protein |  | [27] |
| 2IF1 | 910 | translation initiation factor eif1 |  | [28] |
| 2JSN | 562 | protein transport |  | [29] |
| 2JXC | 1368 | endocytosis |  | [30] |
| 2K21 | 796 | Membrane protein |  | [31] |
| 2K27 | 1517 | transcription regulator |  | [32] |
| 2K3G | 1990 | signaling protein |  | [33] |
| 2KA4 | 961 | transcription regulator |  | [34] |
| 2KA6 | 962 | transcription regulator |  | [34] |
| 2KG4 | 704 | cell cycle |  | [35] |
| 2KMV | 282 | hydrolase |  | [36] |
| 2KQZ | 839 | protein binding |  | [37] |
| 2KR0 | 839 | protein binding |  | [38] |
| 2L3R | 1651 | gene regulation | D | [39] |
| 2L6L | 865 | chaperone | D | [40] |
| 2LGP | 1369 | protein binding |  | [41] |
| 2LY4 | 1493 | anti-tumor protein |  | [42] |
| 2MT6 | 1337 | ligase |  | [43] |
| 2N5X | 1420 | chaperone |  | [44] |
| 2N6F | 887 | heparin-binding protein |  | [45] |
| 2NCA | 1420 | chaperone |  | [46] |
| 2O3D | 1641 | RNA binding |  | [47] |
| 2PRU | 1738 | metal binding |  | [48] |
| 2RN9 | 543 | metal transport |  | [49] |
| 2RTU | 1493 | DNA binding |  | [50] |
| 2UP1 | 324 | gene regulation | D | [51] |
| 2V0O | 2303 | lipid binding |  | [52] |
| 2VWE | 2417 | Immune system |  | [53] |
| 3BZI | 428 | transferase |  | [54] |
| 3CW1 | 1704/2171 | splicing |  | [55] |
| 3KTP | 1324 | protein binding |  | [56] |
| 3RAP | 167 | signaling protein |  | [57] |
| 4BEH | 793 | translation |  | [58] |
| 4HZH | 3299 | hydrolase |  | [59] |
| 4I5N | 1703 | hydrolase |  | [60] |
| 4NZQ | 3299 | hydrolase |  | [61] |
| 4O03 | 3299 | hydrolase |  | [62] |
| 4WQO | 287 | transcription |  | [63] |
| 5FMK | 2540 | apoptosis |  | [64] |
| 5IXF | 1872 | signaling |  | [65] |
| 5JLJ | 1364 | protein transport |  | [66] |
| 5SZW | 2564 | RNA binding |  | [67] |
| 6FZW | 2622 | structural protein |  | [68] |
| 6RPU | 3297 | oxidoreductase |  | [69] |
| 2L42 | 20 | [*Saccharomyces cerevisiae*](https://pl.wikipedia.org/wiki/Caenorhabditis_elegans) |  | [70] |
| 2RNM |  | *Podospora anserina. Fungi.* |  | [71] |

**S2 Table.** The values of parameter RD and of optimal parameter K corresponding to the smallest value $D_{KL}(\mathrm{O}|\mathrm{M})$ (calculated in FOD-M model) for a structural unit (chain/domain) and IDR according to the segmentation shown in Figure 1B (main article). Positions given in bold – the upper subset (green points in Figure 1B), italics – lower subset (red points in Figure 1B). The remaining elements – correspond to orange points in Figure 1B.

| PDB ID | IDR | | RD | | K | | domain |
| --- | --- | --- | --- | --- | --- | --- | --- |
|  | start | fin | chain | IDR | chain | IDR |  |
| 1FHT_A | 102 | 117 | 0.360 | 0.790 | 0.1 | 2.5 |  |
| 2BID_A | 1 | 12 | 0.482 | 0.906 | 0.3 | 2.3 |  |
| 2O3D_A | 196 | 215 | 0.439 | 0.823 | 0.3 | 1.5 |  |
| 2RTU_A | 1 | 13 | 0.457 | 0.782 | 0.3 | 2.5 |  |
| 2RN9_A | 1 | 17 | 0.607 | 0.922 | 0.7 | 2.5 |  |
| 2KQZ_A | 253 | 285 | 0.610 | 0.895 | 0.4 | 1.5 |  |
| **1Z1M_A** | **1** | **24** | **0.520** | **0.803** | **0.3** | **1.3** |  |
| **5SZW_A** | **1** | **19** | **0.421** | **0.668** | **0.2** | **0.7** |  |
| **1UAD_A** | **72** | **83** | **0.379** | **0.621** | **0.2** | **0.8** |  |
| **1F9X_A** | **241** | **254** | **0.595** | **0.833** | **0.5** | **1.6** |  |
| **2BID_A** | **43** | **78** | **0.482** | **0.720** | **0.3** | **1.0** |  |
| **2IF1_A** | **1** | **28** | **0.526** | **0.757** | **0.4** | **1.1** |  |
| **2KG4_A** | **105** | **118** | **0.335** | **0.565** | **0.1** | **0.4** |  |
| **2KMV_A** | **1125** | **1176** | **0.487** | **0.697** | **0.3** | **0.9** |  |
| **2EYW_A** | **121** | **133** | **0.612** | **0.818** | **0.5** | **2.5** |  |
| **2KR0_A** | **1** | **21** | **0.686** | **0.890** | **0.7** | **2.5** |  |
| **2L3R_A** | **165** | **181** | **0.497** | **0.693** | **0.4** | **1.0** |  |
| **2N5X_A** | **343** | **378** | **0.546** | **0.737** | **0.4** | **0.9** |  |
| **2K3G_A** | **104** | **120** | **0.542** | **0.733** | **0.4** | **0.7** |  |
| **1R8U_A** | **258** | **269** | **0.565** | **0.756** | **0.6** | **0.6** |  |
| **2RN9_A** | **1** | **29** | **0.607** | **0.794** | **0.7** | **2.3** |  |
| **3RAP_R** | **45** | **55** | **0.402** | **0.587** | **0.2** | **0.6** |  |
| **2KG4_A** | **1** | **16** | **0.335** | **0.518** | **0.1** | **0.3** |  |
| **2L6L_A** | **1** | **10** | **0.623** | **0.801** | **0.6** | **1.9** | **1-90** |
| **1LXL_A** | **28** | **80** | **0.699** | **0.876** | **0.8** | **2.5** |  |
| **1PC2_A** | **121** | **145** | **0.667** | **0.842** | **0.8** | **0.8** |  |
| **5FMK_B** | **69** | **86** | **0.370** | **0.542** | **0.1** | **0.3** |  |
| **2KA6_B** | **710** | **719** | **0.628** | **0.795** | **0.7** | **0.9** |  |
| **4BEH_A** | **63** | **115** | **0.634** | **0.788** | **0.4** | **1.2** |  |
| **2D3V_A** | **147** | **157** | **0.491** | **0.643** | **0.3** | **0.5** | **96-195** |
| **2KR0_A** | **132** | **252** | **0.686** | **0.835** | **0.7** | **1.4** |  |
| **3CW1_C** | **85** | **97** | **0.412** | **0.559** | **0.2** | **0.8** |  |
| **4HZH_B** | **187** | **211** | **0.537** | **0.672** | **0.5** | **2.5** |  |
| **1F9X_A** | **345** | **356** | **0.595** | **0.724** | **0.5** | **1.0** |  |
| **6FZW_A** | **220** | **233** | **0.602** | **0.730** | **0.5** | **0.6** |  |
| **1D7Q_A** | **124** | **144** | **0.503** | **0.629** | **0.4** | **0.4** |  |
| **1EOT_A** | **1** | **31** | **0.445** | **0.568** | **0.3** | **0.5** |  |
| **4O03_A** | **301** | **316** | **0.444** | **0.567** | **0.1** | **0.5** | **(249-331)+**  **(439-579)** |
| **1DZ7_A** | **59** | **87** | **0.493** | **0.615** | **0.4** | **0.4** |  |
| **2L3R_ A** | **165** | **181** | **0.534** | **0.656** | **0.4** | **0.7** | **125-217** |
| **2L6L_A** | **1** | **10** | **0.641** | **0.762** | **1.3** | **2.5** |  |
| **1P22_B** | **19** | **44** | **0.512** | **0.632** | **0.4** | **0.5** |  |
| **4O03_A** | **301** | **316** | **0.447** | **0.506** | **0.3** | **0.3** |  |
| **3KTP_B** | **1385** | **1398** | **0.502** | **0.612** | **0.2** | **0.4** |  |
| **1DZ7_A** | **33** | **57** | **0.493** | **0.596** | **0.4** | **0.8** |  |
| **4HZH_B** | **169** | **333** | **0.537** | **0.631** | **0.5** | **1.0** |  |
| **3BZI_A** | **489** | **506** | **0.446** | **0.54** | **0.3** | **0.4** | **413-508** |
| **2V0O-B** | **259** | **274** | **0.612** | **0.703** | **0.5** | **1.1** |  |
| **2LGP_A** | **163** | **175** | **0.641** | **0.731** | **0.8** | **1.1** |  |
| **4I5N_A** | **70** | **90** | **0.714** | **0.798** | **1.4** | **2.0** |  |
| **1OQY_A** | **79** | **160** | **0.829** | **0.910** | **2.5** | **2.5** |  |
| **2PRU_A** | **18** | **28** | **0.373** | **0.449** | **0.1** | **0.2** |  |
| **1NI2_A** | **130** | **150** | **0.398** | **0.452** | **0.2** | **0.3** | **83-200** |
| **5JLJ_A** | **33** | **44** | **0.561** | **0.614** | **0.5** | **1.3** |  |
| **2KR0_A** | **386** | **407** | **0.686** | **0.739** | **0.7** | **2.2** |  |
| **2LY4_A** | **1** | **12** | **0.547** | **0.599** | **0.5** | **0.5** |  |
| **1XPA_A** | **168** | **179** | **0.559** | **0.608** | **0.6** | **0.6** |  |
| **1UAD_A** | **72** | **83** | **0.299** | **0.336** | **0.1** | **0.1** |  |
| **2N6F_A** | **32** | **46** | **0.659** | **0.686** | **1.0** | **0.8** | **13-58** |
| **5IXF_A** | **228** | **241** | **0.273** | **0.296** | **0.0** | **0.0** |  |
| *2JXC_B* | *301* | *340* | *0.476* | *0.476* | *0.3* | *0.3* |  |
| *4O03* | *312* | *316* | *0.444* | *0.437* | *0.1* | *0.2* | *(249-331)+*  *(439-579)* |
| *2MT6_A* | *136* | *145* | *0.484* | *0.472* | *0.3* | *0.2* |  |
| *2NCA_A* | *1* | *26* | *0.740* | *0.725* | *2.0* | *2.5* |  |
| *5JLJ_A* | *170* | *198* | *0.561* | *0.539* | *0.5* | *0.6* |  |
| *1RXR_A* | *169* | *189* | *0.370* | *0.345* | *0.1* | *0.0* |  |
| *4HZH_B* | *298* | *316* | *0.537* | *0.511* | *0.5* | *0.4* |  |
| *1R4M_A* | *405* | *463* | *0.670* | *0.637* | *1.1* | *0.9* |  |
| *2K21_A* | *72* | *91* | *0.558* | *0.518* | *0.7* | *0.3* |  |
| *2LY4_A* | *2* | *13* | *0.547* | *0.506* | *0.5* | *0.3* |  |
| *4HZH_B* | *187* | *211* | *0.429* | *0.386* | *0.2* | *0.2* | *169-254* |
| *2L6L_A* | *77* | *92* | *0.641* | *0.596* | *1.3* | *0.8* |  |
| *4HZH-B* | *169* | *333* | *0.654* | *0.606* | *0.8* | *0.9* |  |
| *2D3V_A* | *147* | *157* | *0.687* | *0.633* | *0.8* | *0.4* |  |
| *2K27_A* | *1* | *27* | *0.762* | *0.706* | *2.5* | *1.8* | *1-77* |
| *1TXI_A* | *120* | *164* | *0.619* | *0.560* | *0.7* | *0.6* |  |
| *6FZW-A* | *189* | *200* | *0.602* | *0.541* | *0.5* | *0.3* |  |
| *1UEC_A* | *213* | *228* | *0.707* | *0.644* | *1.2* | *2.3* |  |
| *4NZQ_A* | *301* | *316* | *0.608* | *0.545* | *0.8* | *0.4* |  |
| *2JSN_A* | *11* | *25* | *0.420* | *0.354* | *0.2* | *0.0* |  |
| *2KA4_B* | *786* | *816* | *0.614* | *0.549* | *0.6* | *0.5* |  |
| *2K27* | *135* | *146* | *0.762* | *0.696* | *2.5* | *0.7* |  |
| *3BZI_A* | *489* | *506* | *0.510* | *0.438* | *0.4* | *0.2* |  |
| *2KA6_B* | *720* | *750* | *0.628* | *0.552* | *0.7* | *0.4* |  |
| *6FZW_A* | *151* | *169* | *0.602* | *0.507* | *0.5* | *0.3* | *5-245* |
| *4O03_A* | *301* | *316* | *0.605* | *0.506* | *0.7* | *0.3* |  |
| *1RXR_A* | *178* | *187* | *0.370* | *0.265* | *0.1* | *0.0* |  |
| *1SOH_A* | *40* | *65* | *0.608* | *0.503* | *0.7* | *0.3* |  |
| *2KQ2_A* | *253* | *285* | *0.686* | *0.574* | *0.7* | *0.7* |  |
| *6FZW_A* | *151* | *196* | *0.602* | *0.474* | *0.5* | *0.2* |  |
| *2K21_A* | *107* | *129* | *0.558* | *0.426* | *0.7* | *0.2* |  |
| *1O9U_A* | *285* | *299* | *0.546* | *0.408* | *0.5* | *0.2* | *136-384* |
| *5JLJ_A* | *123* | *144* | *0.561* | *0.421* | *0.5* | *0.1* | *9-216* |
| *1OQY_A* | *283* | *316* | *0.829* | *0.689* | *2.5* | *1.1* |  |
| *2K21_A* | *24* | *45* | *0.558* | *0.417* | *0.7* | *0.1* |  |
| *1B3J_A* | *175* | *184* | *0.729* | *0.583* | *1.5* | *1.3* |  |
| *2KA4_B* | *817* | *838* | *0.614* | *0.465* | *0.6* | *0.3* |  |
| *2D5R_A* | *116* | *138* | *0.466* | *0.314* | *0.4* | *0.0* |  |
| *2VWE_A* | *37* | *46* | *0.544* | *0.387* | *0.5* | *0.1* |  |
| *2K27_A* | *62* | *86* | *0.762* | *0.604* | *2.5* | *0.6* |  |
| *1XPA_A* | *206* | *218* | *0.559* | *0.396* | *0.6* | *0.0* |  |
| *1R4M_A* | *405* | *463* | *0.647* | *0.480* | *0.8* | *0.3* | *388-534* |
| *4O03_A* | *312* | *316* | *0.605* | *0.436* | *0.7* | *0.1* |  |
| *3CW1_K* | *85* | *97* | *0.763* | *0.579* | *0.5* | *0.2* |  |
| *1ZZA_A* | *34* | *59* | *0.632* | *0.439* | *0.5* | *0.1* |  |
| 1OWA_A | 1 | 20 | 0.705 | 0.505 | 0.9 | 0.4 |  |
| 3CW1_E | 85 | 97 | 0.489 | 0.241 | 0.3 | 0.0 |  |
| 1OQY_A | 201 | 231 | 0.829 | 0.564 | 2.5 | 0.4 |  |
| 1DZ7_A | 1 | 10 | 0.493 | 0.224 | 0.4 | 0.0 |  |
| 2UP1_A | 92 | 102 | 0.570 | 0.214 | 0.6 | 0.0 |  |
| 6RPU_A | 27 | 37 | 0.674 | 0.291 | 0.8 | 0.1 |  |

**General characteristics of the *Mus Musculus* IDPs based on the FOD-M model**

The subset of 26 proteins (shown in Table S3) derived from the *Mus Musculus* present in the DisProt database has been analyzed. The reasons for limitation of the whole set are the same as for human protein analysis (see main article). On the basis of the obtained results from FOD-M model (Table S4), it is possible to define their characteristics.

**Figure S1.** The **s**catterplot of the values of parameter *RD* for IDR and its structural unit (chain/domain) for the analyzed whole set of IDPs from Table S3 (two outstanding points denoted by red circle removed from analysis).

The **s**catterplot of the values of the calculated parameter *RD* for the IDR and its structural unit (chain/domain) is presented in Figure S1. Looking at the scatterplot, an approximately linear relationship between the two parameters *RD* can be observed.

The Pearson correlation coefficient (after eliminating the two outstanding points – representing 2BBU and 1DDB proteins – the proteins with much different status) is equal to **0.643**, and the slope of the linear regression – **0.97**.

This means that these proteins appear to be similar to those from the Homo Sapiens (discussed in the main article).

The two specific cases of *Mus Musculus* proteins (denoted by red circles in Figure S1) removed from the analysis due to the significantly different status from others are: Cytokine regulator (2BBU - SH2 domain of murine SOCS3) and a protein involved in apoptosis (1DDB – BID proapoptotic BCL2 family member). The structures of both proteins are characterized by the presence of the long fragments with disorder (34 aa and 40 aa respectively), with a total chain length of 156 aa and 195 aa, which is about 30% of the total chain length. In the scale of values of the RD and K parameters, the fragments with disorder show a really different character compared to the whole molecule. The values of parameter RD for the molecule without IDR segment are **0.475 (K=0.3) and 0.384 (K=0.2) for 2BBU and 1DDB respectively**. This means the high stability of the protein molecule resulting from the presence of a correctly ordered hydrophobic core in the part outside the IDR segment.

These two proteins are unique due to the fact that they account for a very large (much higher than for the others) proportion of disorder. In Figure S2 these fragments are marked as a long red loops. This long, protruding loop does not apply to a 3D gauss distribution which forms the basis of FOD model.


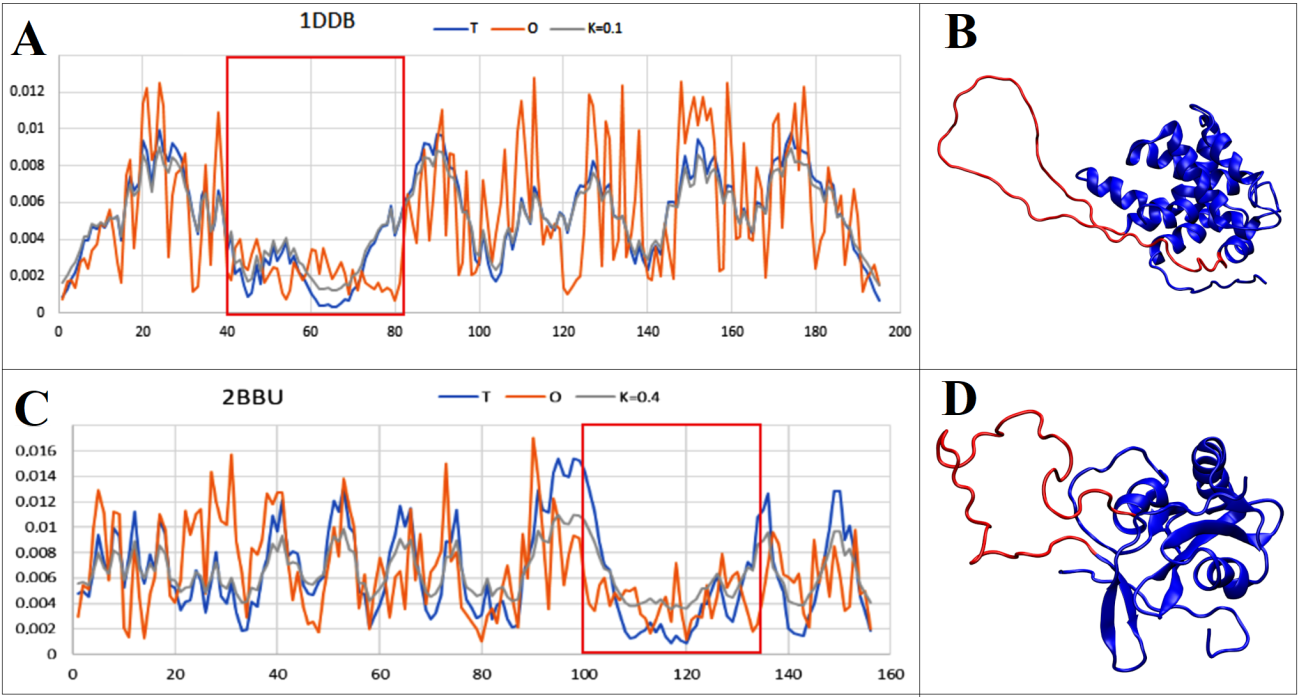


Figure S2. Characteristics of the two outstanding proteins from the Mus Musculus group. The frames distinguish sections with IDR status:

A - 1DDB - profiles T, O and M for K = 0.1,

B - 3D presentation of 1DDB with denoted in red fragment framed in Figure A.

C - 2BBU - profiles T, O and M for K = 0.4;

D - 3D presentation of 2BBU with denoted in red fragment framed in Figure C.

**S3 Table.** List of *Mus Musculus* proteins under consideration from DisProt database with the disordered fragments as present in the structure available in PDB. Column DisProt ID gives the identification number according to DisProt database. D – domain treated as structural unit – all others – chain is treated as a structural unit.

| PDB ID | DisProt ID  DPxxxxx | Functional characteristics | domain | ref |
| --- | --- | --- | --- | --- |
| 2JV3 | 00111 | Transcription |  | [72] |
| 1HN3 | 00335 | Antitumor protein |  | [73] |
| 1CBR-A | 00340 | Retinoic-acid transport |  | [74] |
| 1CBI-A | 00340 | Retinoic-acid transport |  | [75] |
| 3CF2-A | 00435 | Transport protein | D | [76] |
| 2BBU-A | 00446 | Cytokine regulator |  | [77] |
| 2K7M | 00646 | Membrane protein |  | [78] |
| 1U89 | 00656 | Structural protein |  | [79] |
| 4F3L-A | 00734 | Transcription/activator | D | [80] |
| 1V06 | 01323 | DNA binding |  | [81] |
| 1S5R-A | 01323 | Transcription |  | [82] |
| 5NZR-D | 01657 | Transport protein |  | [] |
| 1DDB | 01661 | Apoptosis |  | [83] |
| 2C1M-A | 01879 | Membrane protein |  | [84] |
| 2ZXX-B | 02412 | Cell cycle – replication |  | [85] |
| 6FDD-A | 02426 | Structural protein |  | [86] |
| 3JUA-D | 02451 | Transcription |  | [87] |
| 3UTM-C | 02456 | Transferase/signaling |  | [88] |
| 2D1L-A | 02569 | Protein binding |  | [89] |
| 5OSQ-A | 02586 | Cell adhesion |  | [90] |
| 5DLQ-B | 02593 | Protein transport |  | [91] |
| 1XDK-B | 02633 | Retinoic acid receptor |  |  |
| 5KY3-A | 02659 | Coagulation factor |  | [92] |
| 5KY7-A | 02659 | Coagulation factor |  | [92] |
| 5KY4-A | 02659 | Coagulation factor |  | [92] |
| 5ZO2-A | 02679 | Cell adhesion |  | [93] |
| 2F3J | 03043 | Transport protein | D | [94] |
| 1E7N-A | 03198 | Structural |  | [95] |

**S4 Table.** The values of parameter *RD* and of optimal parameter *K* corresponding to the smallest value $D_{KL}(\mathrm{O}|\mathrm{M})$ (calculated in FOD-M model) for a structural unit (chain/domain) and IDR .

| PDB ID | IDR | | RD | | K | | domain |
| --- | --- | --- | --- | --- | --- | --- | --- |
|  | start | fin | chain | IDR | chain | IDR |  |
| 2JV3 | 29 | 49 | 0.477 | 0.648 | 0.3 | 0.7 |  |
| 1HN3 | 4 | 40 | 0.599 | 0.536 | 0.6 | 0.4 |  |
| 1CBR-A | 1 | 137 | 0.694 | 0.694 | 1.3 | 1.3 |  |
| 1CBI-A | 1 | 137 | 0.670 | 0.670 | 1.1 | 1.1 |  |
| 3CF2-A | 469 | 482 | 0.783 | 0.811 | 2.5 | 1.0 | D |
| 2BBU-A | 100 | 134 | 0.523 | 0.827 | 0.4 | 1.7 |  |
| 2K7M | 2 | 106 | 0.851 | 0.839 | 2.5 | 2.5 |  |
| 1U89 | 799 | 814 | 0.406 | 0.248 | 0.2 | 0.0 |  |
| 4F3L-A | 89 | 107 | 0.642 | 0.695 | 0.8 | 1.5 | D |
| 1V06 | 242 | 285 | 0.477 | 0.520 | 0.3 | 0.4 |  |
| 1S5R-A | 358 | 380 | 0.272 | 0.272 | 0.1 | 0.1 |  |
| 1DDB | 41 | 81 | 0.392 | 0.856 | 0.1 | 2.5 |  |
| 2C1M-A | 47 | 109 | 0.709 | 0.491 | 1.3 | 0.4 |  |
| 2ZXX-B | 79 | 89 | 0.767 | 0.536 | 1.7 | 0.5 |  |
| 6FDD-A | 420 | 430 | 0.691 | 0.603 | 0.9 | 0.5 |  |
| 3JUA-D | 35 | 64 | 0.488 | 0.328 | 0.3 | 0.1 |  |
| 3UTM-C | 19 | 30 | 0.637 | 0.603 | 0.9 | 1.0 |  |
| 2D1L-A | 155 | 168 | 0.733 | 0.526 | 1.3 | 0.4 |  |
| 5OSQ-A | 449 | 461 | 0.716 | 0.872 | 1.5 | 1.3 |  |
| 5DLQ-B | 761 | 771 | 0.700 | 0.352 | 1.5 | 0.1 |  |
| 5KY3 | 225 | 267 | 0.647 | 0.795 | 0.9 | 1.7 |  |
| 5KY7-A | 255 | 267 | 0.652 | 0.728 | 1.0 | 1.3 |  |
| 5KY4-A | 255 | 267 | 0.650 | 0.762 | 1.0 | 1.5 |  |
| 5ZO2-A | 25 | 120 | 0.824 | 0.738 | 1.9 | 1.9 |  |
| 2F3J | 19 | 74 | 0.704 | 0.649 | 1.2 | 1.0 | D |
| 1E7N | 1 | 14 | 0.531 | 0.331 | 0.5 | 0.2 |  |

References

1. Gao C, Ma C, Wang H, Zhong H, Zang J, Zhong R, He F, Yang D. Intrinsic disorder in protein domains contributes to both organism complexity and clade-specific functions. *Sci Rep***2021** ; 11(1):2985.doi: 10.1038/s41598-021-82656-9.
2. Wang H, Zhong H, Gao C, Zang J, Yang D. The Distinct Properties of the Consecutive Disordered Regions Inside or Outside Protein Domains and Their Functional Significance. *Int J Mol Sci***2021** ; 22(19):10677.doi: 10.3390/ijms221910677.
3. Dubreuil B, Matalon O, Levy ED. Protein Abundance Biases the Amino Acid Composition of Disordered Regions to Minimize Non-functional Interactions. *J Mol Biol* **2019** ; 431(24):4978-4992.doi: 10.1016/j.jmb.2019.08.008.
4. Teilum K, Olsen JG, Kragelund BB. On the specificity of protein-protein interactions in the context of disorder. *Biochem J.***2021** ; 478(11):2035-2050 doi: 10.1042/BCJ20200828.
5. Arya S, Singh AK, Bhasne K, Dogra P, Datta A, Das P, Mukhopadhyay S. Femtosecond Hydration Map of Intrinsically Disordered α-Synuclein. *Biophys J***2018** ;114(11):2540-2551.doi: 10.1016/j.bpj.2018.04.028.
6. Dułak D, Gadzała M, Banach M, Konieczny L, Roterman I. Alternative Structures of α-Synuclein. *Molecules.***2020** ;*25(3):*600. doi: 10.3390/molecules25030600.
7. Li P, Willie ST, Bauer S, Morris DL, Spies T, Strong RK. Crystal structure of the MHC class I homolog MIC-A, a gammadelta T cell ligand. *Immunity* **1999**;*10(5):*577-84. doi: 10.1016/s1074-7613(00)80057-6.
8. Battiste JL, Pestova TV, Hellen CU, Wagner G. The eIF1A solution structure reveals a large RNA-binding surface important for scanning function. *Mol Cell* **2000** ; *5(1):*109-19. doi: 10.1016/s1097-2765(00)80407-4.
9. Erbel PJ, Karimi-Nejad Y, De Beer T, Boelens R, Kamerling JP, Vliegenthart JF. Solution structure of the alpha-subunit of human chorionic gonadotropin. *Eur J Biochem* **1999** ;*260(2):*490-8. doi: 10.1046/j.1432-1327.1999.00188.x.
10. Crump MP, Rajarathnam K, Kim KS, Clark-Lewis I, Sykes BD. Solution structure of eotaxin, a chemokine that selectively recruits eosinophils in allergic inflammation. *J Biol Chem* **1998**; *273(35):*22471-9. doi: 10.1074/jbc.273.35.22471.
11. Sun C, Cai M, Meadows RP, Xu N, Gunasekera AH, Herrmann J, Wu JC, Fesik SW. NMR structure and mutagenesis of the third Bir domain of the inhibitor of apoptosis protein XIAP. *J Biol Chem* **2000** ;*275(43):*33777-81. doi: 10.1074/jbc.M006226200.
12. Avis JM, Allain FH, Howe PW, Varani G, Nagai K, Neuhaus D. Solution structure of the N-terminal RNP domain of U1A protein: the role of C-terminal residues in structure stability and RNA binding. *J Mol Biol* **1996** ;*257(2):* 398-411. doi: 10.1006/jmbi.1996.0171.
13. Muchmore SW, Sattler M, Liang H, Meadows RP, Harlan JE, Yoon HS, Nettesheim D, Chang BS, Thompson BC, Wong SL, Ng SL, Fesik SW. X-ray and NMR structure of human Bcl-xL, an inhibitor of programmed cell death. *Nature* **1996** ; *381(6580):*335-41. doi: 10.1038/381335a0.
14. Smith WJ, Nassar N, Bretscher A, Cerione RA, Karplus PA.Structure of the active N-terminal domain of Ezrin. Conformational and mobility changes identify keystone interactions*. J Biol Chem* **2003** ; *278(7):*4949-56. doi: 10.1074/jbc.M210601200.
15. Dajani R, Fraser E, Roe SM, Yeo M, Good VM, Thompson V, Dale TC, Pearl LH. Structural basis for recruitment of glycogen synthase kinase 3beta to the axin-APC scaffold complex. *EMBO J***2003**; *22(3):*494-501. doi: 10.1093/emboj/cdg068.
16. Walters KJ, Lech PJ, Goh AM, Wang Q, Howley PM. DNA-repair protein hHR23a alters its protein structure upon binding proteasomal subunit S5a*Proc Natl Acad Sci U S A.***2003** ; *100(22):*12694-9. doi: 10.1073/pnas.1634989100.
17. Park S, Caffrey MS, Johnson ME, Fung LW-M. Solution structural studies on human erythrocyte alpha-spectrin tetramerization site. *J Biol Chem* **2003** ; *278(24):*21837-44. doi: 10.1074/jbc.M300617200.
18. Wu G, Xu G, Schulman BA, Jeffrey PD, Harper JW, PavletichNP.Structure of a beta-TrCP1-Skp1-beta-catenin complex: destruction motif binding and lysine specificity of the SCF(beta-TrCP1) ubiquitin ligase. *Mol Cell* **2003** ; *11(6):*1445-56. doi: 10.1016/s1097-2765(03)00234-x.
19. Suzuki M, Jeong SY, Karbowski M, Youle RJ, TjandraN.The solution structure of human mitochondria fission protein Fis1 reveals a novel TPR-like helix bundle. *J Mol Biol* **2003** ; *334(3):*445-58. doi: 10.1016/j.jmb.2003.09.064.
20. Walden H, Michael S, Podgorski MS, Huang DT, Miller DW, Howard RJ, Minor DL Jr, Holton JM, Schulman BA.The structure of the APPBP1-UBA3-NEDD8-ATP complex reveals the basis for selective ubiquitin-like protein activation by an E1. *Mol Cell* **2003** ; *12(6):*1427-37. doi: 10.1016/s1097-2765(03)00452-0.
21. De Guzman RN, Martinez-Yamout MA, Dyson HJ, Wright PE Interaction of the TAZ1 domain of the CREB-binding protein with the activation domain of CITED2: regulation by competition between intrinsically unstructured ligands for non-identical binding sites. *J Biol Chem* **2004** ;*279(4):*3042-9. doi: 10.1074/jbc.M310348200.
22. Holmbeck SM, Foster MP, Casimiro DR, Sem DS, Dyson HJ, Wright PE. High-resolution solution structure of the retinoid X receptor DNA-binding domain. *J Mol Biol* **1998** ;*281(2):*271-84. doi: 10.1006/jmbi.1998.1908.
23. MacRaild CA, Howlett GJ, Gooley PR. The structure and interactions of human apolipoprotein C-II in dodecyl phosphocholine. *Biochemistry* **2004** ;*43(25):*8084-93. doi: 10.1021/bi049817l
24. Eelen G, Verlinden L, Rochel N, Claessens F, De Clercq P, Vandewalle M, Tocchini-Valentini G, Moras D, Bouillon R, Verstuyf A. Superagonistic action of 14-epi-analogs of 1,25-dihydroxyvitamin D explained by vitamin D receptor-coactivator interaction. *Mol Pharmacol* **2005**; *67(5):*1566-73. doi: 10.1124/mol.104.008730.
25. Fukai S, Matern HT, Jagath JR, Scheller RH, Brunger AT. Structural basis of the interaction between RalA and Sec5, a subunit of the sec6/8 complex. *EMBO J***2003** ;*22(13):*3267-78. doi: 10.1093/emboj/cdg329.
26. Yuzawa S, Suzuki NN, Fujioka Y, Ogura K, Sumimoto H, Inagaki F. A molecular mechanism for autoinhibition of the tandem SH3 domains of p47phox, the regulatory subunit of the phagocyte NADPH oxidase. *Genes Cells***2004** ;*9(5):*443-56. doi: 10.1111/j.1356-9597.2004.00733.x.
27. Ikegami T, Kuraoka I, Saijo M, Kodo N, Kyogoku Y, Morikawa K, Tanaka K, Shirakawa M.Solution structure of the DNA- and RPA-binding domain of the human repair factor XPA. *Nat Struct Biol* **1998** ;*5(8):*701-6. doi: 10.1038/1400
28. Uhrinova S, Uhrin D, Powers H, Watt K, Zheleva D, Fischer P, McInnes C, Barlow PN. Structure of free MDM2 N-terminal domain reveals conformational adjustments that accompany p53-binding. *J Mol Biol***2005**; *350(3):*587-98. doi: 10.1016/j.jmb.2005.05.010.
29. Buck-Koehntop BA, Mascioni A, Buffy JJ, Veglia G. Structure, dynamics, and membrane topology of stannin: a mediator of neuronal cell apoptosis induced by trimethyltin chloride. Structure, dynamics, and membrane topology of stannin: a mediator of neuronal cell apoptosis induced by trimethyltin chloride. *J Mol Biol***2005** ;*354(3*):652-65. doi: 10.1016/j.jmb.2005.09.038.
30. Chou JJ, Li H, Salvesen GS, Yuan J, Wagner G. Solution structure of BID, an intracellular amplifier of apoptotic signaling. *Cell***1999** ;*96(5):*615-24. doi: 10.1016/s0092-8674(00)80572-3
31. Shiroishi M, Kajikawa M, Kuroki K, Ose T, Kohda D, MaenakaK.Crystal structure of the human monocyte-activating receptor, "Group 2" leukocyte Ig-like receptor A5 (LILRA5/LIR9/ILT11). *J Biol Chem***2006** ;*281(28*): 19536-44. doi: 10.1074/jbc.M603076200
32. Horiuchi M, Takeuchi K, Noda N, Muroya N, Suzuki T, Nakamura T, Kawamura-Tsuzuku J, Takahasi K, Yamamoto T, Inagaki F. Structural basis for the antiproliferative activity of the Tob-hCaf1 complex. *J Biol Chem***2009**; *284(19)*: 13244-55. doi: 10.1074/jbc.M809250200
33. Kobashigawa Y, Sakai M, Naito M, Yokochi M, Kumeta H, Makino Y, Ogura K, Tanaka S, Inagaki F. Structural basis for the transforming activity of human cancer-related signaling adaptor protein CRK *Nat Struct Mol Biol***2007** ;*14(6):* 503-10. doi: 10.1038/nsmb1241
34. Fletcher CM, Pestova TV, Hellen CU, Wagner G. Structure and interactions of the translation initiation factor eIF1. *EMBO J***1999** ;*18(9):* 2631-7. doi: 10.1093/emboj/18.9.2631.
35. Fan S, Feng Y, Wei Z, Xia B, Gong W. Solution structure of synbindin atypical PDZ domain and interaction with syndecan-2. *Protein Pept Lett***2009**; *16(2):* 189-95. doi: 10.2174/092986609787316342
36. Rumpf J, Simon B, Jung N, Maritzen T, Haucke V, Sattler M, Groemping Y. Structure of the Eps15-stonin2 complex provides a molecular explanation for EH-domain ligand specificity. *EMBO J***2008**; *27(3):*558-69. doi: 10.1038/sj.emboj.7601980.
37. Kang C, Tian C, Sönnichsen FD, Smith JA, Meiler J, George AL Jr, Vanoye CG, Kim HJ, Sanders CR. Structure of KCNE1 and implications for how it modulates the KCNQ1 potassium channel. *Biochemistry***2008** ;*47(31):*7999-8006. doi: 10.1021/bi800875q
38. Codutti L, van Ingen H, Vascotto C, Fogolari F, Corazza A, Tell G, Quadrifoglio F, Viglino P, Boelens R, Esposito G. The solution structure of DNA-free Pax-8 paired box domain accounts for redox regulation of transcriptional activity in the pax protein family. *J Biol Chem***2008** ;*283(48):* 33321-8.doi: 10.1074/jbc.M805717200.
39. Klages J, Kotzsch A, Coles M, Sebald W, Nickel J, Müller T, Kessler H. The solution structure of BMPR-IA reveals a local disorder-to-order transition upon BMP-2 binding. *Biochemistry***2008** ;*47(46):* 11930-9. doi: 10.1021/bi801059j.
40. Wojciak JM, Martinez-Yamout MA, Dyson HJ, Wright PE. Structural basis for recruitment of CBP/p300 coactivators by STAT1 and STAT2 transactivation domains. *EMBO J***2009** ;*28(7):* 948-58. doi: 10.1038/emboj.2009.30.
41. Sánchez R, Pantoja-Uceda D, Prieto J, Diercks T, Marcaida MJ, Montoya G, Campos-Olivas R, Blanco FJ. Solution structure of human growth arrest and DNA damage 45alpha (Gadd45alpha) and its interactions with proliferating cell nuclear antigen (PCNA) and Aurora A kinase. *J Biol Chem***2010**; *285(29):* 22196-201. doi: 10.1074/jbc.M109.069344.
42. Banci L, Bertini I, Cantini F, Inagaki S, Migliardi M, Rosato A. The binding mode of ATP revealed by the solution structure of the N-domain of human ATP7A. *J Biol Chem* **2010** ;*285(4):* 2537-44. doi: 10.1074/jbc.M109.054262.
43. Chen X, Lee B-H, Finley D, Walters KJ. Structure of proteasome ubiquitin receptor hRpn13 and its activation by the scaffolding protein hRpn2. Mol Cell **2010** ; 38(3):404-15.doi: 10.1016/j.molcel.2010.04.019.
44. Chen X, Lee BH, Finley D, Walters KJ. Structure of proteasome ubiquitin receptor hRpn13 and its activation by the scaffolding protein hRpn2. *Mol Cell***2010**, *38*:404-415.
45. Nady N, Lemak A, Walker RJ, Avvakumov GV, Kareta MS, Achour M, Xue S, Duan S, Allali-Hassani A, Zuo X, Wang YX, Bronner C, Chédin F, Arrowsmith CH, Dhe-Paganon S. Recognition of multivalent histone states associated with heterochromatin by UHRF1 protein. *J Biol Chem***2011**, *286*:24300-24311
46. Thakur A, Chitoor B, Goswami AV, Pareek G, Atreya HS, D'Silva P. Structure and mechanistic insights into novel iron-mediated moonlighting functions of human J-protein cochaperone, Dph4. *J Biol Chem***2012**, *287:*13194-13205
47. Guttman M, Komives EA. The structure, dynamics, and binding of the LA45 module pair of the low-density lipoprotein receptor suggest an important role for LA4 in ligand release. *Biochemistry* **2011***, 50:* 11001-11008
48. Rowell JP, Simpson KL, Stott K, Watson M, Thomas JO. HMGB1-facilitated p53 DNA binding occurs via HMG-Box/p53 transactivation domain interaction, regulated by the acidic tail. *Structure***2012**, *20*: 2014-2024.
49. Vittal V, Shi L, Wenzel DM, Scaglione KM, Duncan ED, Basrur V, Elenitoba-Johnson KS, Baker D, Paulson HL, Brzovic PS, Klevit RE. Intrinsic disorder drives N-terminal ubiquitination by Ube2w. *Nat Chem Biol***2015**, *11*:83-89 doi: 10.1038/nchembio.1700
50. Zhang Z, Keramisanou D, Dudhat A, Paré M, Gelis I. The C-terminal domain of human Cdc37 studied by solution NMR. *BiomolNmr***2015**, *63:*315-321. 10.1007/s10858-015-9988-6
51. Ryan E, Shen D, Wang X. Structural studies reveal an important role for the pleiotrophin C-terminus in mediating interactions with chondroitin sulfate. *Febs J***2016**, *283*:1488-1503 Doi: 10.1111/febs.13686
52. Keramisanou D, Aboalroub A, Zhang Z, Liu W, Marshall D, Diviney A, Larsen RW, Landgraf R, Gelis I. Molecular Mechanism of Protein Kinase Recognition and Sorting by the Hsp90 Kinome-Specific Cochaperone Cdc37. *Mol Cell***2016**, *62:*260-271. doi: 10.1016/j.molcel.2016.04.005
53. Tintaru AM, Hautbergue GM, Hounslow AM, Hung ML, Lian LY, Craven CJ, Wilson SA. Structural and functional analysis of RNA and TAP binding to SF2/ASF. *EMBO Rep***2007**, *8:*756-762. doi: 10.1038/sj.embor.7401031
54. Malik S, Revington M, Smith SP, Shaw GS. Analysis of the structure of human apo-S100B at low temperature indicates a unimodal conformational distribution is adopted by calcium-free S100 proteins. *Proteins***2008**, *73:*28-42 , doi: 10.1002/prot.22037
55. Banci L, Bertini I, Ciofi-Baffoni S, Janicka A, Martinelli M, Kozlowski H, Palumaa P. A structural-dynamical characterization of human Cox17. *J Biol Chem***2008**, *283:*7912-7920. DOI: 10.1074/jbc.M708016200
56. Wang J, Tochio N, Takeuchi A, Uewaki J, Kobayashi N, Tate S. Redox-sensitive structural change in the A-domain of HMGB1 and its implication for the binding to cisplatin modified DNA. *BiochemBiophys Res Commun***2013**, *441:*701-706.
57. Ding J, Hayashi MK, Zhang Y, Manche L, Krainer AR, Xu RM. Crystal structure of the two-RRM domain of hnRNP A1 (UP1) complexed with single-stranded telomeric DNA. *Genes Dev***1999**, *13:*1102-1115.
58. Henne WM, Kent HM, Ford MG, Hegde BG, Daumke O, Butler PJ, Mittal R, Langen R, Evans PR, McMahon HT. Structure and analysis of FCHo2 F-BAR domain: a dimerizing and membrane recruitment module that effects membrane curvature. *Structure***2007**, *15:*839-852 DOI: 10.1016/j.str.2007.05.002
59. Leonard P, Scotney PD, Jabeen T, Iyer S, Fabri LJ, Nash AD, Acharya KR. Crystal structure of vascular endothelial growth factor-B in complex with a neutralising antibody Fab fragment. *J Mol Biol***2008**, *384:*1203-1217 DOI: 10.1016/j.jmb.2008.09.076
60. García-Alvarez B, de Cárcer G, Ibañez S, Bragado-Nilsson E, Montoya G. Molecular and structural basis of polo-like kinase 1 substrate recognition: Implications in centrosomal localization. *Proc Natl Acad Sci U S A***2007**, *104:* 3107-3112 DOI: 10.1073/pnas.0609131104
61. Pomeranz-Krummel DA, Oubridge C, Leung AK, Li J, Nagai K. Crystal structure of human spliceosomal U1 snRNP at 5.5 A resolution. *Nature***2009**, *458:* 475-480. DOI: 10.1038/nature07851
62. Kozlov G, Safaee N, Rosenauer A, Gehring K. Structural basis of binding of P-body-associated proteins GW182 and ataxin-2 by the Mlle domain of poly(A)-binding protein. *J Biol Chem***2010**, *285:* 13599-13606.
63. Ménétrey J, Cherfils J. Structure of the small G protein Rap2 in a non-catalytic complex with GTP. *Proteins***1999**, *37:* 465-473, DOI: 10.1002/(SICI)1097-0134(19991115)37:3<465::AID-PROT13>3.0.CO;2-O
64. Sauvage D, Souvage E, Herman R, Kerff M, Rocaboy M, Charlier P. Crystal structures of r39-Imipenem acyl-Enzyme - PDB
65. Pozzi N, Chen Z, Gohara DW, Niu W, Heyduk T, Di Cera E. Crystal structure of prothrombin reveals conformational flexibility and mechanism of activation. *Biol Chem***2013**, *288:* 22734-22744 DOI: 10.1074/jbc.M113.466946J
66. Wlodarchak N, Guo F, Satyshur KA, Jiang L, Jeffrey PD, Sun T, Stanevich V, Mumby MC, Xing Y. Structure of the Ca2+-dependent PP2A heterotrimer and insights into Cdc6 dephosphorylation. *Cell Res***2013**, *23:*931-946 doi: 10.1038/cr.2013.77
67. Pozzi N, Chen Z, Pelc LA, Shropshire DB, Di Cera E. The linker connecting the two kringles plays a key role in prothrombin activation. *Proc Natl Acad Sci U S A***2014** ; 111(21):7630-5.doi: 10.1073/pnas.1403779111.
68. Pozzi N, Chen Z, Pelc LA, Shropshire DB, Di Cera E. The linker connecting the two kringles plays a key role in prothrombin activation. *Proc Natl Acad Sci U S A***2014**, *111:*7630-7635 doi: 10.1073/pnas.1403779111
69. Nguyen HC, Yang H, Fribourgh JL, Wolfe LS, Xiong Y. Insights into Cullin-RING E3 ubiquitin ligase recruitment: structure of the VHL-EloBC-Cul2 complex. *Structure***2015**, *23*: 441-449, DOI : 10.1016/j.str.2014.12.014
70. 112, Lee EF, Grabow S, Chappaz S, Dewson G, Hockings C, Kluck RM, Debrincat MA, Gray DH, Witkowski MT, Evangelista M, Pettikiriarachchi A, Bouillet P, Lane RM, Czabotar PE, Colman PM, Smith BJ, Kile BT, Fairlie WD. Physiological restraint of Bak by Bcl-xL is essential for cell survival. *Genes Dev***2016**, *30:*1240-1250 DOI: 10.1101/gad.279414.116
71. Wasmer C, Lange A, Van Melckebeke H, Siemer AB, Riek R, Meier BH. Amyloid fibrils of the HET-s(218-289) prion form a beta solenoid with a triangular hydrophobic core. Science 2008 ; 319(5869):1523-6.doi: 10.1126/science.1151839.
72. 2JV3 Lee GM, Kang H, Schaerpf M, Slupsky CM, Lawrence MP. Ets-1pnt domain (29-138) nmr structure ensamble. PDB
73. 1HN3 [DiGiammarino](https://pubmed.ncbi.nlm.nih.gov/?term=DiGiammarino+EL&cauthor_id=11327858) EL, [Filippov](https://pubmed.ncbi.nlm.nih.gov/?term=Filippov+I&cauthor_id=11327858) I, [Weber](https://pubmed.ncbi.nlm.nih.gov/?term=Weber+JD&cauthor_id=11327858) JD, [Bothner](https://pubmed.ncbi.nlm.nih.gov/?term=Bothner+B&cauthor_id=11327858) B, [Kriwacki](https://pubmed.ncbi.nlm.nih.gov/?term=Kriwacki+RW&cauthor_id=11327858) RW. Solution structure of the p53 regulatory domain of the p19Arf tumor suppressor protein. Biochemistry 2001 ;40(8):2379-86. doi: 10.1021/bi0024005.
74. 1CBR [Kleywegt](https://pubmed.ncbi.nlm.nih.gov/?term=Kleywegt+GJ&cauthor_id=7704533) GJ, [Bergfors](https://pubmed.ncbi.nlm.nih.gov/?term=Bergfors+T&cauthor_id=7704533) T, [Senn](https://pubmed.ncbi.nlm.nih.gov/?term=Senn+H&cauthor_id=7704533) H, [Le Motte](https://pubmed.ncbi.nlm.nih.gov/?term=Le+Motte+P&cauthor_id=7704533) P, [Gsell](https://pubmed.ncbi.nlm.nih.gov/?term=Gsell+B&cauthor_id=7704533) B, [Shudo](https://pubmed.ncbi.nlm.nih.gov/?term=Shudo+K&cauthor_id=7704533) K, [Jones](https://pubmed.ncbi.nlm.nih.gov/?term=Jones+TA&cauthor_id=7704533) TA. Crystal structures of cellular retinoic acid binding proteins I and II in complex with all-trans-retinoic acid and a synthetic retinoid. Structure 1994;2(12):1241-58. doi: 10.1016/s0969-2126(94)00125-1.
75. 1CBI [Thompson](https://pubmed.ncbi.nlm.nih.gov/?term=Thompson+JR&cauthor_id=7563063) JR, [Bratt](https://pubmed.ncbi.nlm.nih.gov/?term=Bratt+JM&cauthor_id=7563063) JM, [Banaszak](https://pubmed.ncbi.nlm.nih.gov/?term=Banaszak+LJ&cauthor_id=7563063) LJ. Crystal structure of cellular retinoic acid binding protein I shows increased access to the binding cavity due to formation of an intermolecular beta-sheet. J Mol Biol 1995 ;252(4):433-46. doi: 10.1006/jmbi.1995.0509.
76. 3CF2 [Davies](https://pubmed.ncbi.nlm.nih.gov/?term=Davies+JM&cauthor_id=18462676) JM, [Brunger](https://pubmed.ncbi.nlm.nih.gov/?term=Brunger+AT&cauthor_id=18462676) AT, [Weis](https://pubmed.ncbi.nlm.nih.gov/?term=Weis+WI&cauthor_id=18462676) WI. Improved structures of full-length p97, an AAA ATPase: implications for mechanisms of nucleotide-dependent conformational change. Structure 2008 , 16(5):715-26. doi: 10.1016/j.str.2008.02.010.

1. [Babon](https://pubmed.ncbi.nlm.nih.gov/?term=Babon+JJ&cauthor_id=16630890) JJ, [McManus](https://pubmed.ncbi.nlm.nih.gov/?term=McManus+EJ&cauthor_id=16630890) EJ, [Yao](https://pubmed.ncbi.nlm.nih.gov/?term=Yao+S&cauthor_id=16630890) S, [DeSouza](https://pubmed.ncbi.nlm.nih.gov/?term=DeSouza+DP&cauthor_id=16630890) DP, [Mielke](https://pubmed.ncbi.nlm.nih.gov/?term=Mielke+LA&cauthor_id=16630890) LA, [Sprigg](https://pubmed.ncbi.nlm.nih.gov/?term=Sprigg+NS&cauthor_id=16630890) NS, [Willson](https://pubmed.ncbi.nlm.nih.gov/?term=Willson+TA&cauthor_id=16630890) TA, [Hilton](https://pubmed.ncbi.nlm.nih.gov/?term=Hilton+DJ&cauthor_id=16630890) DJ, [Nicola](https://pubmed.ncbi.nlm.nih.gov/?term=Nicola+NA&cauthor_id=16630890) NA, [Baca](https://pubmed.ncbi.nlm.nih.gov/?term=Baca+M&cauthor_id=16630890) M, [Nicholson](https://pubmed.ncbi.nlm.nih.gov/?term=Nicholson+SE&cauthor_id=16630890) SE, [Norton](https://pubmed.ncbi.nlm.nih.gov/?term=Norton+RS&cauthor_id=16630890) RS. The structure of SOCS3 reveals the basis of the extended SH2 domain function and identifies an unstructured insertion that regulates stability. Mol Cell 2006 ;22(2):205-16. doi: 10.1016/j.molcel.2006.03.024.

1. [Bouvier](https://pubmed.ncbi.nlm.nih.gov/?term=Bouvier+D&cauthor_id=19808665) D, [Spagnol](https://pubmed.ncbi.nlm.nih.gov/?term=Spagnol+G&cauthor_id=19808665) G, [Chenavas](https://pubmed.ncbi.nlm.nih.gov/?term=Chenavas+S&cauthor_id=19808665) S, [Kieken](https://pubmed.ncbi.nlm.nih.gov/?term=Kieken+F&cauthor_id=19808665) F, [Vitrac](https://pubmed.ncbi.nlm.nih.gov/?term=Vitrac+H&cauthor_id=19808665) H, [Brownell](https://pubmed.ncbi.nlm.nih.gov/?term=Brownell+S&cauthor_id=19808665) S, [Kellezi](https://pubmed.ncbi.nlm.nih.gov/?term=Kellezi+A&cauthor_id=19808665) A, [Forge](https://pubmed.ncbi.nlm.nih.gov/?term=Forge+V&cauthor_id=19808665) V, [Sorgen](https://pubmed.ncbi.nlm.nih.gov/?term=Sorgen+PL&cauthor_id=19808665) PL. Characterization of the structure and intermolecular interactions between the connexin40 and connexin43 carboxyl-terminal and cytoplasmic loop domains. J Biol Chem 2009 ;284(49):34257-71. doi: 10.1074/jbc.M109.039594.

1. [Fillingham](https://pubmed.ncbi.nlm.nih.gov/?term=Fillingham+I&cauthor_id=15642262) I, [Gingras](https://pubmed.ncbi.nlm.nih.gov/?term=Gingras+AR&cauthor_id=15642262) AR, [Papagrigoriou](https://pubmed.ncbi.nlm.nih.gov/?term=Papagrigoriou+E&cauthor_id=15642262) E, [Patel](https://pubmed.ncbi.nlm.nih.gov/?term=Patel+B&cauthor_id=15642262) B, [Emsley](https://pubmed.ncbi.nlm.nih.gov/?term=Emsley+J&cauthor_id=15642262) J, [Critchley](https://pubmed.ncbi.nlm.nih.gov/?term=Critchley+DR&cauthor_id=15642262) DR, [Roberts](https://pubmed.ncbi.nlm.nih.gov/?term=Roberts+GC&cauthor_id=15642262) GCK, [Barsukov](https://pubmed.ncbi.nlm.nih.gov/?term=Barsukov+IL&cauthor_id=15642262) IL. A vinculin binding domain from the talin rod unfolds to form a complex with the vinculin head. Structure 2005 ;13(1):65-74. doi: 10.1016/j.str.2004.11.006.

1. [Huang](https://pubmed.ncbi.nlm.nih.gov/?term=Huang+N&cauthor_id=22653727) N, [Chelliah](https://pubmed.ncbi.nlm.nih.gov/?term=Chelliah+Y&cauthor_id=22653727) Y, [Shan](https://pubmed.ncbi.nlm.nih.gov/?term=Shan+Y&cauthor_id=22653727) Y, [Taylor](https://pubmed.ncbi.nlm.nih.gov/?term=Taylor+CA&cauthor_id=22653727) CA, [Yoo](https://pubmed.ncbi.nlm.nih.gov/?term=Yoo+SH&cauthor_id=22653727) S-H, [Partch](https://pubmed.ncbi.nlm.nih.gov/?term=Partch+C&cauthor_id=22653727) C, [Green](https://pubmed.ncbi.nlm.nih.gov/?term=Green+CB&cauthor_id=22653727) CB, [Zhang](https://pubmed.ncbi.nlm.nih.gov/?term=Zhang+H&cauthor_id=22653727) H, [Takahashi](https://pubmed.ncbi.nlm.nih.gov/?term=Takahashi+JS&cauthor_id=22653727) JS. Crystal structure of the heterodimeric CLOCK:BMAL1 transcriptional activator complex. Science 2012 ;337(6091):189-94. doi: 10.1126/science.1222804.

1. [de Chiara](https://pubmed.ncbi.nlm.nih.gov/?term=de+Chiara+C&cauthor_id=15893665) C, [Menon](https://pubmed.ncbi.nlm.nih.gov/?term=Menon+RP&cauthor_id=15893665) RP, [Adinolfi](https://pubmed.ncbi.nlm.nih.gov/?term=Adinolfi+S&cauthor_id=15893665) S, [de Boer](https://pubmed.ncbi.nlm.nih.gov/?term=de+Boer+J&cauthor_id=15893665) J, [Ktistaki](https://pubmed.ncbi.nlm.nih.gov/?term=Ktistaki+E&cauthor_id=15893665) E, [Kelly](https://pubmed.ncbi.nlm.nih.gov/?term=Kelly+G&cauthor_id=15893665) G, [Calder](https://pubmed.ncbi.nlm.nih.gov/?term=Calder+L&cauthor_id=15893665) L, [Dimitris Kioussis](https://pubmed.ncbi.nlm.nih.gov/?term=Kioussis+D&cauthor_id=15893665) D, [Pastore](https://pubmed.ncbi.nlm.nih.gov/?term=Pastore+A&cauthor_id=15893665) A. The AXH domain adopts alternative folds the solution structure of HBP1 AXH. Structure 2005 ;13(5):743-53. doi: 10.1016/j.str.2005.02.016.

1. [Swanson](https://pubmed.ncbi.nlm.nih.gov/?term=Swanson+KA&cauthor_id=15235594) KA, [Knoepfler](https://pubmed.ncbi.nlm.nih.gov/?term=Knoepfler+PS&cauthor_id=15235594) PS, [Huang](https://pubmed.ncbi.nlm.nih.gov/?term=Huang+K&cauthor_id=15235594) K, [Kang](https://pubmed.ncbi.nlm.nih.gov/?term=Kang+RS&cauthor_id=15235594) RS, [Cowley](https://pubmed.ncbi.nlm.nih.gov/?term=Cowley+SM&cauthor_id=15235594) SM, [Laherty](https://pubmed.ncbi.nlm.nih.gov/?term=Laherty+CD&cauthor_id=15235594) CD, [Eisenman](https://pubmed.ncbi.nlm.nih.gov/?term=Eisenman+RN&cauthor_id=15235594) RN, [Radhakrishnan](https://pubmed.ncbi.nlm.nih.gov/?term=Radhakrishnan+I&cauthor_id=15235594) I. HBP1 and Mad1 repressors bind the Sin3 corepressor PAH2 domain with opposite helical orientations. Nat Struct Mol Biol 2004 ;11(8):738-46.doi: 10.1038/nsmb798.

1. [McDonnell](https://pubmed.ncbi.nlm.nih.gov/?term=McDonnell+JM&cauthor_id=10089878) JM, [Fushman](https://pubmed.ncbi.nlm.nih.gov/?term=Fushman+D&cauthor_id=10089878) D, [Milliman](https://pubmed.ncbi.nlm.nih.gov/?term=Milliman+CL&cauthor_id=10089878) CL, [Korsmeyer](https://pubmed.ncbi.nlm.nih.gov/?term=Korsmeyer+SJ&cauthor_id=10089878) SJ, [Cowburn](https://pubmed.ncbi.nlm.nih.gov/?term=Cowburn+D&cauthor_id=10089878) D. Solution structure of the proapoptotic molecule BID: a structural basis for apoptotic agonists and antagonists. Cell 1999 ;96(5):625-34. doi: 10.1016/s0092-8674(00)80573-5.

1. [Matsuura](https://pubmed.ncbi.nlm.nih.gov/?term=Matsuura+Y&cauthor_id=16222336) Y, [Stewart](https://pubmed.ncbi.nlm.nih.gov/?term=Stewart+M&cauthor_id=16222336) M. Nup50/Npap60 function in nuclear protein import complex disassembly and importin recycling. EMBO J 2005 ;24(21):3681-9. doi: 10.1038/sj.emboj.7600843.

1. [Lee](https://pubmed.ncbi.nlm.nih.gov/?term=Lee+C&cauthor_id=15286659) C, [Hong](https://pubmed.ncbi.nlm.nih.gov/?term=Hong+B&cauthor_id=15286659) B-S, [Choi](https://pubmed.ncbi.nlm.nih.gov/?term=Choi+JM&cauthor_id=15286659) JM, [Kim](https://pubmed.ncbi.nlm.nih.gov/?term=Kim+Y&cauthor_id=15286659) Y, [Watanabe](https://pubmed.ncbi.nlm.nih.gov/?term=Watanabe+S&cauthor_id=15286659) S, [Ishimi](https://pubmed.ncbi.nlm.nih.gov/?term=Ishimi+Y&cauthor_id=15286659) Y, [Enomoto](https://pubmed.ncbi.nlm.nih.gov/?term=Enomoto+T&cauthor_id=15286659) T, [Tada](https://pubmed.ncbi.nlm.nih.gov/?term=Tada+S&cauthor_id=15286659) S, [Kim](https://pubmed.ncbi.nlm.nih.gov/?term=Kim+Y&cauthor_id=15286659) Y, [Cho](https://pubmed.ncbi.nlm.nih.gov/?term=Cho+Y&cauthor_id=15286659) Y. Structural basis for inhibition of the replication licensing factor Cdt1 by geminin. Nature 2004 ; 430(7002):913-7. doi: 10.1038/nature02813.

1. [Delhommel](https://pubmed.ncbi.nlm.nih.gov/?term=Delhommel+F&cauthor_id=30053338) F, [Cordier](https://pubmed.ncbi.nlm.nih.gov/?term=Cordier+F&cauthor_id=30053338) F, [Saul](https://pubmed.ncbi.nlm.nih.gov/?term=Saul+F&cauthor_id=30053338) F, [Chataigner](https://pubmed.ncbi.nlm.nih.gov/?term=Chataigner+L&cauthor_id=30053338) L, [Haouz](https://pubmed.ncbi.nlm.nih.gov/?term=Haouz+A&cauthor_id=30053338) A, [Wolff](https://pubmed.ncbi.nlm.nih.gov/?term=Wolff+N&cauthor_id=30053338) N. Structural plasticity of the HHD2 domain of whirlin. FEBS J 2018 ;285(20):3738-3752. doi: 10.1111/febs.14614.

1. [Chen](https://pubmed.ncbi.nlm.nih.gov/?term=Chen+L&cauthor_id=20123908) L, [Chan](https://pubmed.ncbi.nlm.nih.gov/?term=Chan+SW&cauthor_id=20123908) SW, [Zhang](https://pubmed.ncbi.nlm.nih.gov/?term=Zhang+X&cauthor_id=20123908) XQ, [Walsh](https://pubmed.ncbi.nlm.nih.gov/?term=Walsh+M&cauthor_id=20123908) M, [Lim](https://pubmed.ncbi.nlm.nih.gov/?term=Lim+CJ&cauthor_id=20123908) CY, [Hong](https://pubmed.ncbi.nlm.nih.gov/?term=Hong+W&cauthor_id=20123908) W, [Song](https://pubmed.ncbi.nlm.nih.gov/?term=Song+H&cauthor_id=20123908) H. Structural basis of YAP recognition by TEAD4 in the hippo pathway. Genes Dev 2010 ;24(3):290-300. doi: 10.1101/gad.1865310.

1. [Morrone](https://pubmed.ncbi.nlm.nih.gov/?term=Morrone+S&cauthor_id=22307604) S, [Cheng](https://pubmed.ncbi.nlm.nih.gov/?term=Cheng+Z&cauthor_id=22307604) Z, [Moon](https://pubmed.ncbi.nlm.nih.gov/?term=Moon+RT&cauthor_id=22307604) RT, [Cong](https://pubmed.ncbi.nlm.nih.gov/?term=Cong+F&cauthor_id=22307604) F, [Xu](https://pubmed.ncbi.nlm.nih.gov/?term=Xu+W&cauthor_id=22307604) W. Crystal structure of a Tankyrase-Axin complex and its implications for Axin turnover and Tankyrase substrate recruitment. Proc Natl Acad Sci U S A 2012 ;109(5):1500-5. doi: 10.1073/pnas.1116618109.

1. [Lee](https://pubmed.ncbi.nlm.nih.gov/?term=Lee+SH&cauthor_id=17292833) SH, [Kerff](https://pubmed.ncbi.nlm.nih.gov/?term=Kerff+F&cauthor_id=17292833) F, [Chereau](https://pubmed.ncbi.nlm.nih.gov/?term=Chereau+D&cauthor_id=17292833) D, [Ferron](https://pubmed.ncbi.nlm.nih.gov/?term=Ferron+F&cauthor_id=17292833) F, [Klug](https://pubmed.ncbi.nlm.nih.gov/?term=Klug+A&cauthor_id=17292833) A, [Dominguez](https://pubmed.ncbi.nlm.nih.gov/?term=Dominguez+R&cauthor_id=17292833) R. Structural basis for the actin-binding function of missing-in-metastasis. Structure 2007;15(2):145-55. doi: 10.1016/j.str.2006.12.005.

1. [Monné](https://pubmed.ncbi.nlm.nih.gov/?term=Monn%C3%A9+M&cauthor_id=19052627) M, [Han](https://pubmed.ncbi.nlm.nih.gov/?term=Han+L&cauthor_id=19052627) L, [Schwend](https://pubmed.ncbi.nlm.nih.gov/?term=Schwend+T&cauthor_id=19052627) T, [Burendahl](https://pubmed.ncbi.nlm.nih.gov/?term=Burendahl+S&cauthor_id=19052627) S, [Jovine](https://pubmed.ncbi.nlm.nih.gov/?term=Jovine+L&cauthor_id=19052627) L. Crystal structure of the ZP-N domain of ZP3 reveals the core fold of animal egg coats. Nature 2008 ;456(7222):653-7. doi: 10.1038/nature07599.

1. [Aksu](https://pubmed.ncbi.nlm.nih.gov/?term=Aksu+M&cauthor_id=27306458) M, [Trakhanov](https://pubmed.ncbi.nlm.nih.gov/?term=Trakhanov+S&cauthor_id=27306458) S, [Görlich](https://pubmed.ncbi.nlm.nih.gov/?term=G%C3%B6rlich+D&cauthor_id=27306458) D. Structure of the exportin Xpo4 in complex with RanGTP and the hypusine-containing translation factor eIF5A. Nat Commun 2016 ;7:11952. doi: 10.1038/ncomms11952.

1. [Li](https://pubmed.ncbi.nlm.nih.gov/?term=Li+Z&cauthor_id=28530709) Z, [Han](https://pubmed.ncbi.nlm.nih.gov/?term=Han+K&cauthor_id=28530709) K, [Pak](https://pubmed.ncbi.nlm.nih.gov/?term=Pak+JE&cauthor_id=28530709) JE, [Satkunarajah](https://pubmed.ncbi.nlm.nih.gov/?term=Satkunarajah+M&cauthor_id=28530709) M, [Zhou](https://pubmed.ncbi.nlm.nih.gov/?term=Zhou+D&cauthor_id=28530709) D, [Rini](https://pubmed.ncbi.nlm.nih.gov/?term=Rini+JM&cauthor_id=28530709) JM. Recognition of EGF-like domains by the Notch-modifying O-fucosyltransferase POFUT1. Nat Chem Biol 2017 ;13(7):757-763. doi: 10.1038/nchembio.2381.

1. [Liu](https://pubmed.ncbi.nlm.nih.gov/?term=Liu+X&cauthor_id=30674679) X, [An](https://pubmed.ncbi.nlm.nih.gov/?term=An+T&cauthor_id=30674679) T, [Li](https://pubmed.ncbi.nlm.nih.gov/?term=Li+D&cauthor_id=30674679) D, [Fan](https://pubmed.ncbi.nlm.nih.gov/?term=Fan+Z&cauthor_id=30674679) Z, [Xiang](https://pubmed.ncbi.nlm.nih.gov/?term=Xiang+P&cauthor_id=30674679) P, [Li](https://pubmed.ncbi.nlm.nih.gov/?term=Li+C&cauthor_id=30674679) C, [Ju](https://pubmed.ncbi.nlm.nih.gov/?term=Ju+W&cauthor_id=30674679) W, [Li](https://pubmed.ncbi.nlm.nih.gov/?term=Li+J&cauthor_id=30674679) J, [Hu](https://pubmed.ncbi.nlm.nih.gov/?term=Hu+G&cauthor_id=30674679) G, [Qin](https://pubmed.ncbi.nlm.nih.gov/?term=Qin+B&cauthor_id=30674679) B, [Yin](https://pubmed.ncbi.nlm.nih.gov/?term=Yin+B&cauthor_id=30674679) B, [Wojdyla](https://pubmed.ncbi.nlm.nih.gov/?term=Wojdyla+JA&cauthor_id=30674679) JA, [Wang](https://pubmed.ncbi.nlm.nih.gov/?term=Wang+M&cauthor_id=30674679) M, [Yuan](https://pubmed.ncbi.nlm.nih.gov/?term=Yuan+J&cauthor_id=30674679), [Qiang](https://pubmed.ncbi.nlm.nih.gov/?term=Qiang+B&cauthor_id=30674679) B, [Shu](https://pubmed.ncbi.nlm.nih.gov/?term=Shu+P&cauthor_id=30674679) P, [Cui](https://pubmed.ncbi.nlm.nih.gov/?term=Cui+S&cauthor_id=30674679) S, [Peng](https://pubmed.ncbi.nlm.nih.gov/?term=Peng+X&cauthor_id=30674679) X. Structure of the heterophilic interaction between the nectin-like 4 and nectin-like 1 molecules. Proc Natl Acad Sci USA 2019;116(6):2068-2077. doi: 10.1073/pnas.1810969116.

1. [Golovanov](https://pubmed.ncbi.nlm.nih.gov/?term=Golovanov+AP&cauthor_id=17000901) AP, [Hautbergue](https://pubmed.ncbi.nlm.nih.gov/?term=Hautbergue+GM&cauthor_id=17000901) GM, [Tintaru](https://pubmed.ncbi.nlm.nih.gov/?term=Tintaru+AM&cauthor_id=17000901) AM, [Lian](https://pubmed.ncbi.nlm.nih.gov/?term=Lian+LY&cauthor_id=17000901) L-Y, [Wilson](https://pubmed.ncbi.nlm.nih.gov/?term=Wilson+SA&cauthor_id=17000901) SA. The solution structure of REF2-I reveals interdomain interactions and regions involved in binding mRNA export factors and RNA. RNA 2006 ;12(11):1933-48. doi: 10.1261/rna.212106.

1. [Clout](https://pubmed.ncbi.nlm.nih.gov/?term=Clout+NJ&cauthor_id=11090271) NJ, [Basak](https://pubmed.ncbi.nlm.nih.gov/?term=Basak+A&cauthor_id=11090271) A, [Wieligmann](https://pubmed.ncbi.nlm.nih.gov/?term=Wieligmann+K&cauthor_id=11090271) K, [Bateman](https://pubmed.ncbi.nlm.nih.gov/?term=Bateman+OA&cauthor_id=11090271) OA, [Jaenicke](https://pubmed.ncbi.nlm.nih.gov/?term=Jaenicke+R&cauthor_id=11090271) R, [Slingsby](https://pubmed.ncbi.nlm.nih.gov/?term=Slingsby+C&cauthor_id=11090271) C. The N-terminal domain of betaB2-crystallin resembles the putative ancestral homodimer. J Mol Biol 2000 ;304(3):253-7. doi: 10.1006/jmbi.2000.4197.
